# Molecular Modes of Action of an Aqueous *Nerium oleander* Extract in Cancer Cells In Vitro and In Vivo

**DOI:** 10.3390/molecules28041871

**Published:** 2023-02-16

**Authors:** Luay J. Rashan, Nadire Özenver, Joelle C. Boulos, Mona Dawood, Wynand P. Roos, Katrin Franke, Ioannis Papasotiriou, Ludger A. Wessjohann, Heinz-Herbert Fiebig, Thomas Efferth

**Affiliations:** 1 Frankincense Biodiversity Unit, Research Center, Dhofar University, Salalah 211, Oman; 2 Department of Pharmaceutical Biology, Institute of Pharmaceutical and Biomedical Sciences, Johannes Gutenberg University, 55128 Mainz, Germany; 3 Department of Pharmacognosy, Faculty of Pharmacy, Hacettepe University, Ankara 06100, Turkey; 4 4HF Biotec GmbH, 79108 Freiburg, Germany; 5 Department of Molecular Biology, Faculty of Medical Laboratory Sciences, Al-Neelain University, Khartoum 12702, Sudan; 6 Institute of Toxicology, Medical Center of the University Mainz, Obere Zahlbacher Straße 67, 55131 Mainz, Germany; 7 Department of Bioorganic Chemistry, Leibniz Institute of Plant Biochemistry (IPB), Weinberg 3, 06120 Halle, Germany; 8 Research Genetic Cancer Centre (RGCC) International GmbH, 6300 Zug, Switzerland

**Keywords:** Apocynaceae, cardiac glycoside, cardenolides, mitotic spindle poison, phytotherapy

## Abstract

Cancer drug resistance remains a major obstacle in clinical oncology. As most anticancer drugs are of natural origin, we investigated the anticancer potential of a standardized cold-water leaf extract from *Nerium oleander* L., termed Breastin. The phytochemical characterization by nuclear magnetic resonance spectroscopy (NMR) and low- and high-resolution mass spectrometry revealed several monoglycosidic cardenolides as major constituents (adynerin, neritaloside, odoroside A, odoroside H, oleandrin, and vanderoside). Breastin inhibited the growth of 14 cell lines from hematopoietic tumors and 5 of 6 carcinomas. Remarkably, the cellular responsiveness of odoroside H and neritaloside was not correlated with all other classical drug resistance mechanisms, i.e., ATP-binding cassette transporters (ABCB1, ABCB5, ABCC1, ABCG2), oncogenes (EGFR, RAS), tumor suppressors (TP53, WT1), and others (GSTP1, HSP90, proliferation rate), in 59 tumor cell lines of the National Cancer Institute (NCI, USA), indicating that Breastin may indeed bypass drug resistance. COMPARE analyses with 153 anticancer agents in 74 tumor cell lines of the Oncotest panel revealed frequent correlations of Breastin with mitosis-inhibiting drugs. Using tubulin-GFP-transfected U2OS cells and confocal microscopy, it was found that the microtubule-disturbing effect of Breastin was comparable to that of the tubulin-depolymerizing drug paclitaxel. This result was verified by a tubulin polymerization assay in vitro and molecular docking in silico. Proteome profiling of 3171 proteins in the NCI panel revealed protein subsets whose expression significantly correlated with cellular responsiveness to odoroside H and neritaloside, indicating that protein expression profiles can be identified to predict the sensitivity or resistance of tumor cells to Breastin constituents. Breastin moderately inhibited breast cancer xenograft tumors in vivo. Remarkably, in contrast to what was observed with paclitaxel monotherapy, the combination of paclitaxel and Breastin prevented tumor relapse, indicating Breastin’s potential for drug combination regimens.

## 1. Introduction

*Nerium oleander* L. (Family: Apocynaceae) is a shrub growing in subtropical regions (Mediterranean Basin, Arabian Peninsula, Southwest Asia). It is the only species belonging to the genus *Nerium*. It is an ornamental plant in parks and gardens and has a long history in ancient Europe. In the Near East and Southern Asia, it has also been used in traditional medicine as anti-inflammatory, antidiabetic, and anticancer herbal medicine as well as an herbal drug against indigestion, malaria, leprosy, mental diseases, etc. [1,2,3,4]. It is a medicinal herb in Indian Ayurveda and Unani [1].

However, the plant is also known for its toxicity [5]. Among other phytochemicals, the bioactive molecules of *N. oleander* are cardiac glycosides such as oleandrin, which are toxic upon ingestion and have a narrow therapeutic window. In academic Western medicine, cardiac glycosides have been used to treat heart insufficiency [6]. The positive inotropic effect is mediated by the inhibition of the membrane-integrated Na^+^/K^+^-ATPase (e.g., [7]). The advent of the angiotensin-converting enzyme (ACE) inhibitors replaced the therapeutic use of cardiac glycosides, but in the case of nonresponsiveness to ACE inhibition, cardiac glycosides are still very helpful, if blood serum levels are closely monitored to avoid intoxication [6,7,8,9,10]. Cardiac glycosides also exert profound anticancer activity in vitro and in vivo [10,11,12,13,14,15,16,17,18]. Oleandrin was found to downregulate the DNA damage repair protein Rad51 [19]. Other mechanisms involved in the anticancer cytotoxic activity of *N. oleander* and oleandrin include the inhibition of glycolysis [18].

Even though *N. oleander* is a toxic plant, its profound anticancer activity might nevertheless qualify it as a source for further drug development. Compounds from other highly toxic plants also made their way into clinical oncology. These include vinblastine and vincristine from *Catharanthus roseus* [20], paclitaxel from *Taxus brevifolia* [21], and the semisynthetic derivatives from *Podophyllum peltatum*, namely etoposide and teniposide [22]. Consequently, *N. oleander* should not be prematurely ignored because of its toxicity, especially since in vivo experiments and preliminary clinical trials demonstrated tolerable side effects and acceptable safety profiles. Therefore, the question remains whether *N. oleander* may serve as a source for developing anticancer drugs. 

The present study aimed to investigate the anticancer activity of a cold-water extract of *N. oleander* leaves termed “Breastin” in vitro and in vivo. To confirm a stable and good quality, we determined the chemical constituents of Breastin to verify the constituents that have been previously reported by us. Breastin is distinguished from other extracts (hot-water, alcoholic, cf. our earlier work) in being a cold-water extract [23]. Then, we measured the cytotoxicity of Breastin in a panel of hematopoietic and solid tumor cell lines. To gain insight into the molecular modes of action, we performed *COMPARE* analyses using the National Cancer Institute (USA) and Oncotest panels of cell lines and Breastin as well as two of Breastin’s constituents, odoroside H and neritaloside. We have chosen these two compounds as data were not available for the other phytochemicals of Breastin. The inhibition of the microtubule network was identified as a possible mode of action of Breastin and verified in subsequent experiments. Furthermore, proteomic profiling identified subsets of proteins that predicted the sensitivity or resistance to odoroside H and neritaloside.

## 2. Results

### 2.1. Phytochemical Profiling of Breastin

**Figure 1:**
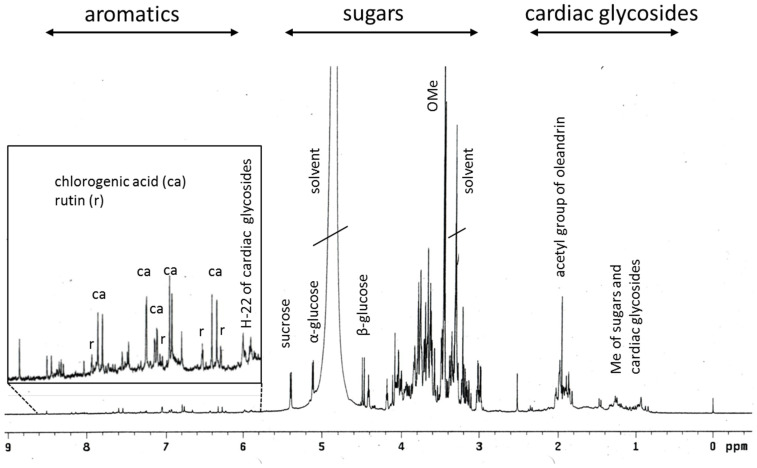
^1^H-NMR profile of Breastin.

**Figure 2:**
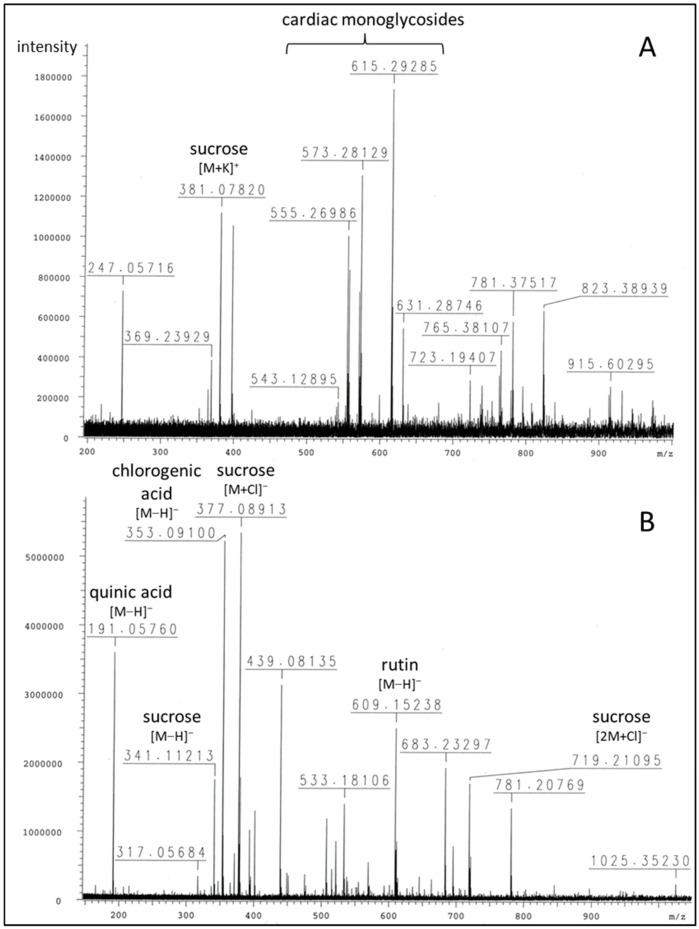
FTICR-HRMS spectra of Breastin in positive (**A**) and negative (**B**) ionization modes (see Table 1 for details).

**Figure 3:**
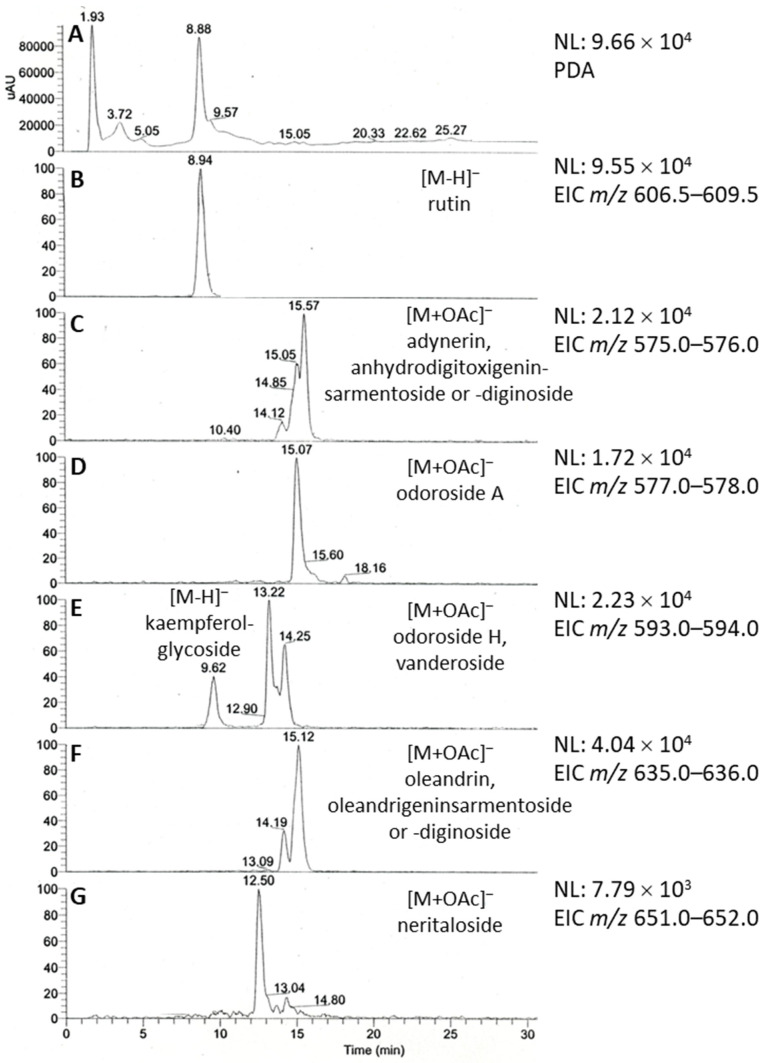
LC/ESI-MS profile of Breastin in negative ionization mode. (**A**) Photodiode array (PDA) absorbance spectrum. (**B**–**G**) Extracted ion chromatograms (EICs) of major secondary metabolites corresponding to the flavonoid glycosides rutin (**B**) and kaempferol rhamnoglucoside (**E**) and cardiac monoglycosides (**C**–**G**).

The chemical composition of the Breastin preparation was investigated by nuclear magnetic resonance spectroscopy (NMR) and low- and high-resolution mass spectrometry. The intension was to confirm the previously published chemoprofile [23] as a measure of quality control of the extraction process and to reveal the composition of the Breastin preparation in addition to cardiac glycosides. The proton NMR spectrum indicates the predominant occurrence of sugars and glycosidic compounds (Figure 1). The major sugars are glucose as well as the disaccharide sucrose, which represents a common storage compound in plants. As expected for aqueous preparations, nonpolar fatty compounds as well as aromatics were underrepresented. Breastin contains the phenolic compounds chlorogenic acid and rutin (Figure 1 and Figure 2). Rutin was already described in 1956 as the major flavonoid glycoside in N. oleander leaves [24]. Rutin is accompanied by kaempferol rhamnoglucoside. The presence of this compound was confirmed by our data ([M−H]- at *m/z* 593, Figure 3). Chlorogenic acid was previously identified as an anti-inflammatory compound in N. oleander flowers [25]. However, the relevant cytotoxic compounds responsible for the anticancer effects are cardiac glycosides. MS data show the occurrence of the active monoglycosidic cardenolides (Figure 2 and Figure 3, Table 1) previously isolated from the Breastin preparation, i.e., oleandrin, oleandrigenin sarmentoside, neritaloside, odoroside H, and odoroside A [23]. These monoglycosidic cardenolides possessing the 3β,14β-dihydroxy-5β-card-20(22)-enolide structure with or without an acetoxy group at C-16 were shown to exhibit significant anticancer activity and might be preferentially extracted (vs. less active genins, di- or triglycosides) by maceration of *N. oleander* leaves with water at room temperature [23].

### 2.2. Inhibition of Cell Viability of Cell Lines of Hematopoietic and Solid Tumor Origins

**Figure 4:**
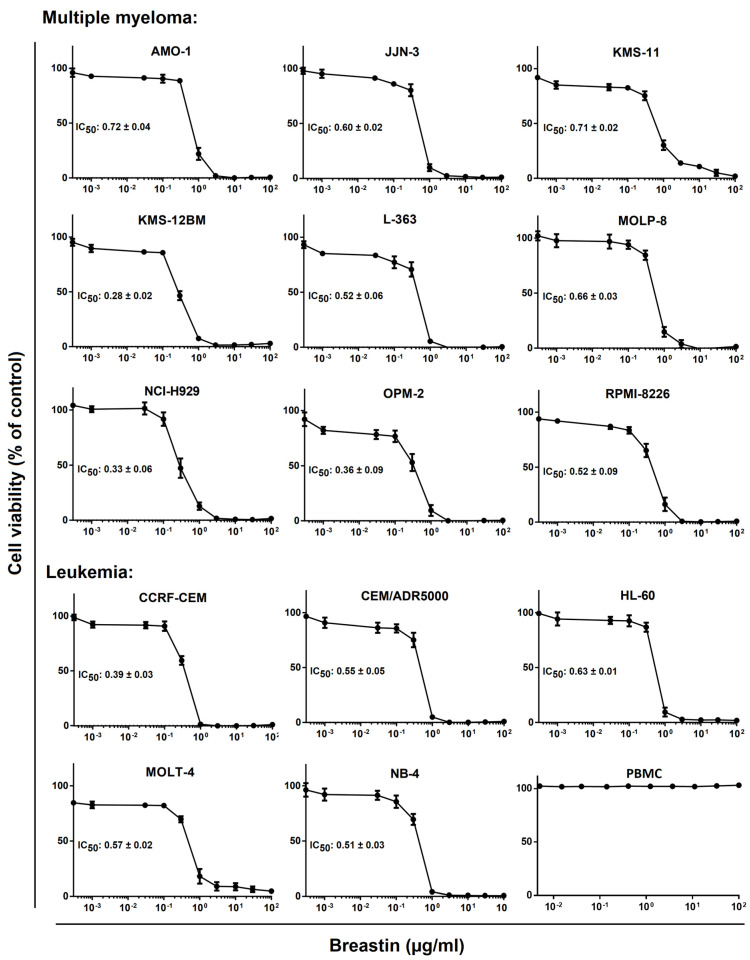
Determination of the in vitro activity of Breastin in human multiple myeloma and leukemia cell lines as measured by the resazurin assay. The upper panel shows the 9 tested multiple myeloma KMS11, KMS12BM, NCI-H929, MolP8, JJN3, OPM2, AMO1, and L363 cell lines, and the lower panel shows the five leukemia cell lines, i.e., CCRF-CEM and MOLT-4 acute lymphoblastic leukemia, NB4 and HL60 acute promyelocytic leukemia cells. CEM/ADR500 cells are a multidrug-resistant subline of CCRF-CEM. The IC_50_ values have been calculated from the dose–response curves. The dose–response curves shown each represent three independent experiments with six parallel measurements each.

The effect of Breastin on the viability of multiple myeloma and leukemia cell lines was analyzed by generating dose–response curves with concentrations ranging from 10^−3^ to 10^2^ µg/mL (Figure 4). The dose–response curves were used to calculate the 50% inhibition concentrations (IC_50_). For the multiple myeloma cell lines, they ranged from 0.28 to 0.72 µg/mL (Figure 4, upper panel). The leukemia cell lines tested showed IC_50_ values in a comparable range (0.39 to 0.63 µg/mL). Based on the IC_50_ values, the multidrug-resistant CEM/ADR5000 subline of CCRF-CEM was not resistant or was only minimally resistant (1.4-fold) to Breastin compared to the parental drug-sensitive cells. This was remarkable since CEM/ADR5000 were characterized as expressing a phenotype of full-blown high-degree multidrug resistance to a broad array of clinically established anticancer drugs, including anthracyclines, Vinca alkaloids, epipodophyllotoxines, taxanes, and others [26]. 

**Figure 5:**
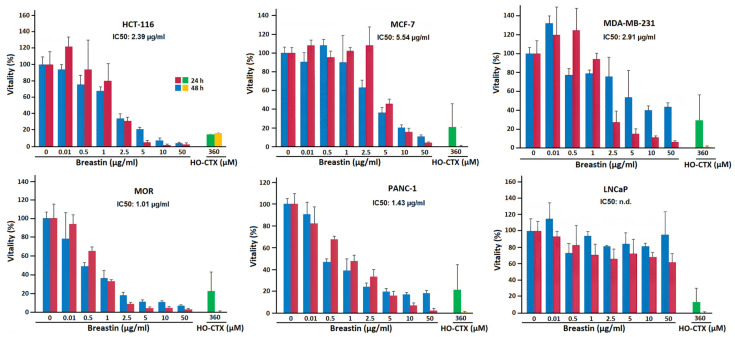
Determination of the in vitro activity of Breastin in various human solid cancer cell lines of the RGCC panel as measured by the MTT assay. We tested HCT-116 colorectal carcinoma, MCF-7 and MDA-MB-231 breast adenocarcinoma, MOR lung adenocarcinoma, PANC-1 pancreatic carcinoma, and LnCaP prostate adenocarcinoma cells. 4-Hydroxycyclophosphamide (HO-CTX) was used as control drug.

For comparison, we also exemplarily tested cell lines derived from solid tumors. The results of six carcinoma cell lines treated with Breastin are shown in Figure 5. In five cell lines, Breastin was active and we obtained IC_50_ values ranging from 1.01 to 5.54 µg/mL. Another cell line (LNCaP) was not responsive to Breastin and an IC_50_ value could not be calculated, even up to the highest concentration of 50 µg/mL. Furthermore, we compared the effects of incubation with Breastin for 24 h and 48 h. We did not observe an increased growth inhibition after 48 h compared to 24 h, indicating that shorter incubation might be sufficient to exert Breastin’s effect.

### 2.3. Role of Classical Drug Resistance Mechanisms for Odoroside H and Neritaloside in the NCI Cell Line Panel

**Figure 6:**
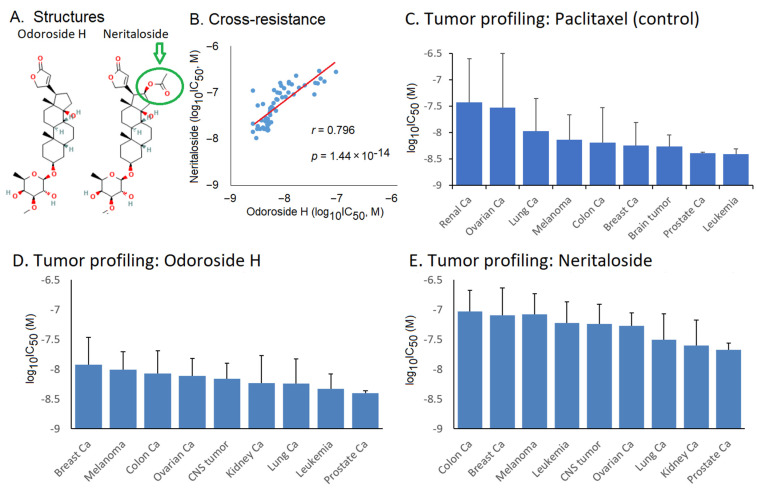
Tumor type and cross-resistance profiling of odoroside H and neritaloside in response to standard anticancer drugs. (**A**) Chemical structures; (**B**) cross-resistance between these two drugs based on their log_10_IC_50_ values in the NCI cell lines; tumor profiling for (**C**) paclitaxel (control drug), (**D**) odoroside H, and (**E**) neritaloside based on the log_10_IC_50_ values of the NCI panel (mean ± SD).

The fact that Breastin inhibited multidrug-resistant CEM/ADR5000 cells with nearly the same efficacy as drug-sensitive CCRF-CEM cells piqued our interest, and we wanted to analyze the activity of Breastin in a panel of classical drug resistance mechanisms in greater detail. Odoroside H and neritaloside as two of the main compounds of Breastin were investigated in the NCI panel of 59 tumor cell lines. The data deposited in the NCI database were used for their evaluation. Both compounds are cardenolides and have the same scaffold, and neritaloside has an additional acetoxy group at position C16 (Figure 6A). The other compounds could not be considered further because they are not deposited in the NCI database (https://dtp.cancer.gov; accessed on 8 February 2023). In the case of oleandrin, its cytotoxicity was beyond the concentration range tested in the NCI panel; therefore, it could not be used further.

We first analyzed whether both compounds are involved in drug resistance phenotypes caused by diverse mechanisms, such as ATP-binding cassette transporters (ABCB1 (MDR1/P-glycoprotein, ABCB5, ABCC1/MRP1, and ABCG2/BCRP), oncogenes and tumor suppressors (EGFR, RAS, TP53, and WT1), and others (glutathione S-transferase π, heat shock protein HSP90, and the proliferation rate of the cell lines) (Table 2). Importantly, we did not observe statistically significant correlations between the responsiveness of these cell lines to the vast majority of the resistance parameters. This indicates that the activity of these two Breastin constituents was not hampered by the major classical anticancer drug resistance mechanisms.

### 2.4. Cross-Resistance Profile of the NCI Cell Line Panel of Standard Anticancer Agents in Response to Odoroside H and Neritaloside

The log_10_IC_50_ values of odoroside H and neritaloside were first correlated with each other. We found a significant relationship (r = 0.798, *p* = 1.44 × 10^−14^) (Figure 6B). The cytotoxicity of odoroside H and neritaloside was comparable to that of paclitaxel, which was used as a control drug (Figure 6C–E). There was a trend that leukemia and prostate carcinoma cell lines were more sensitive to odoroside H than breast cancer and melanoma cell lines and that kidney and prostate carcinoma cell lines were more sensitive to neritaloside than colon and breast cancer cell lines.

### 2.5. COMPARE Analysis of the Oncotest Cell Line Panel of Transcriptome-Wide mRNA Expression in Response to Breastin

In addition to the two isolated compounds, odoroside H and neritaloside, we were also interested in the N. oleander extract itself. We performed COMPARE analyses with Breastin in another panel of cell lines. For this reason, we used the tumor cell line panel of Oncotest GmbH (Freiburg, Germany) and correlated the IC_50_ and IC_70_ values for Breastin of 74 cell lines with 153 reference anticancer agents with known modes of action.

The IC_50_ and IC_70_ of the top-ranked compounds (r > 0.37/0.38, *p* < 0.05) are listed in Table 3. The IC_70_ values have been compiled in addition to the usually used IC_50_ values to have additional information at drug concentrations that are noncytotoxic and which therefore might better reflect the mode of action without the involvement of cytotoxic effects. It was a striking result that the COMPARE profile of Breastin was associated with many anticancer agents affecting mitosis. This can be taken as an indication that mitosis and/or DNA damage-related mechanisms may contribute to the cytotoxic effect of Breastin on cancer cells. Therefore, we independently performed in vitro experiments to prove these biostatistical correlation analyses.

### 2.6. Breastin Affected the Microtubule Network as Detected by Confocal Microscopy

**Figure 7:**
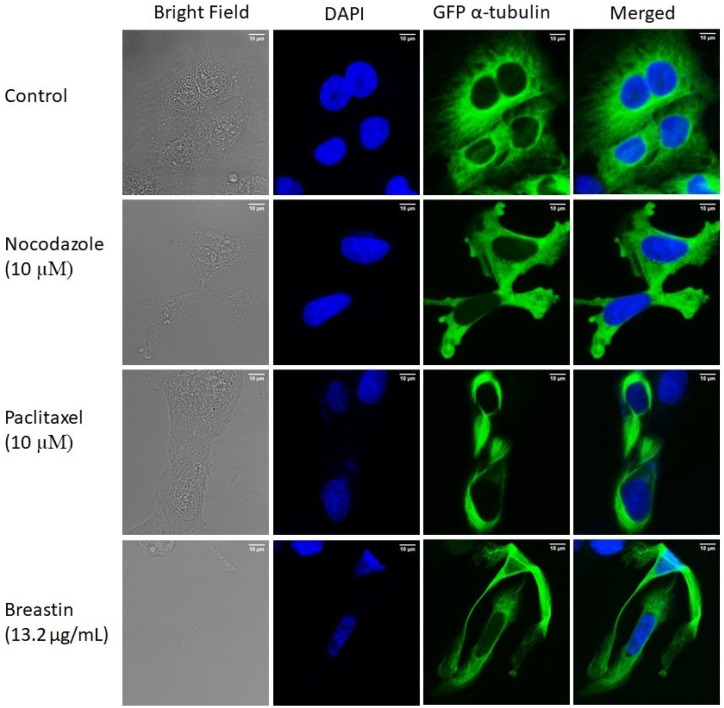
Enhanced microtubule network in Breastin-treated U2OS cells. Micrographs of cells fixed with 4% paraformaldehyde were taken 24 h post-treatment with DMSO, Breastin (13.2 µg/mL), paclitaxel (10 µM), and nocodazole (10 µM). DAPI was used to stain nuclei. Images were taken at 40× magnification (scale bars = 10 µm) with the AF7000 widefield fluorescence microscope.

U2OS human osteosarcoma cells expressing α-tubulin-GFP were treated with Breastin (13.2 µg/mL) for 24 h to study the impact of Breastin on microtubules. Paclitaxel (10 µM) and nocodazole (10 µM) were taken as positive controls. As shown in Figure 7, tubulin was perfectly polymerized in control cells. In fact, tubulin was distributed along the cytoplasm as a thick intracellular network. By contrast, a stiff tubulin network was observed in Breastin-treated cells. This result was comparable to paclitaxel-treated cells. Unlike nocodazole, which extensively disintegrated the tubulin network, Breastin and paclitaxel increased the rigidity of microtubules at the boundaries. These results provide evidence that Breastin, just like paclitaxel, enhances tubulin polymerization.

### 2.7. Breastin Affected Microtubule Polymerization in a Cell-Free Based In Vitro Assay

**Figure 8:**
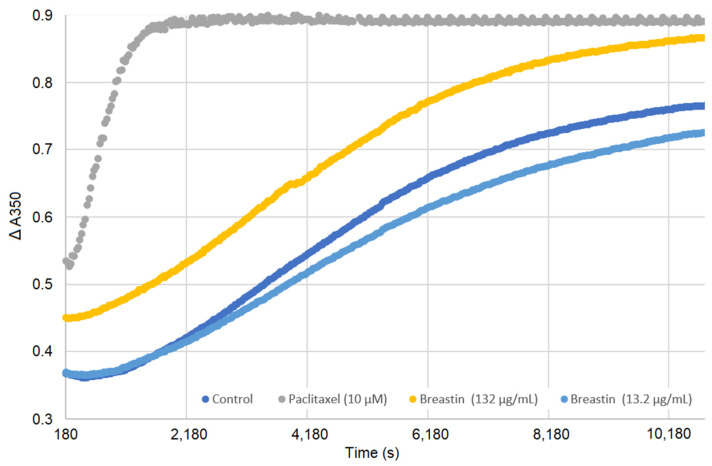
Enhancement of tubulin polymerization by Breastin. Tubulin was incubated with DMSO (negative control), Breastin (13.2 and 132 µg/mL), and paclitaxel (10 µM) at 37 °C. Optical density was determined every 30 s for 3 h. A shift to the upper left compared to DMSO reflected an increase in microtubule polymerization.

An in vitro tubulin polymerization assay was performed to validate the results obtained by confocal microscopy. Paclitaxel, a microtubule-stabilizing agent, was taken as a positive control. As depicted in Figure 8, paclitaxel enhanced microtubule polymerization at high concentrations. At low concentrations, Breastin did not display any significant activity. However, at the higher concentration, an increase in light scattering was observed at 350 nm (comparable to paclitaxel beyond 8000 s), indicating that Breastin enhances tubulin polymerization similarly to paclitaxel.

### 2.8. Molecular Docking of Phytochemicals from Breastin

Our phytochemical analyses (Figure 3) identified several other phytochemicals in addition to odoroside H and neritaloside as the main constituents of Breastin. Therefore, we were interested in whether the other compounds might also interact with tubulin, and we performed molecular docking studies. As a first step, we used the blind docking approach for the α/β-tubulin dimer to see whether there are considerable binding affinities of these compounds at all. As shown in Table 4, the control drugs vinorelbine and paclitaxel showed the lowest binding energies (LBEs). The highest LBE was observed with colchicine (−6.82 kcal/mol). The LBEs of the Breastin constituents were between those of the three control drugs, ranging between −8.06 and −8.80 kcal/mol, indicating that these substances may be interacting with tubulin or microtubules. 

**Figure 9:**
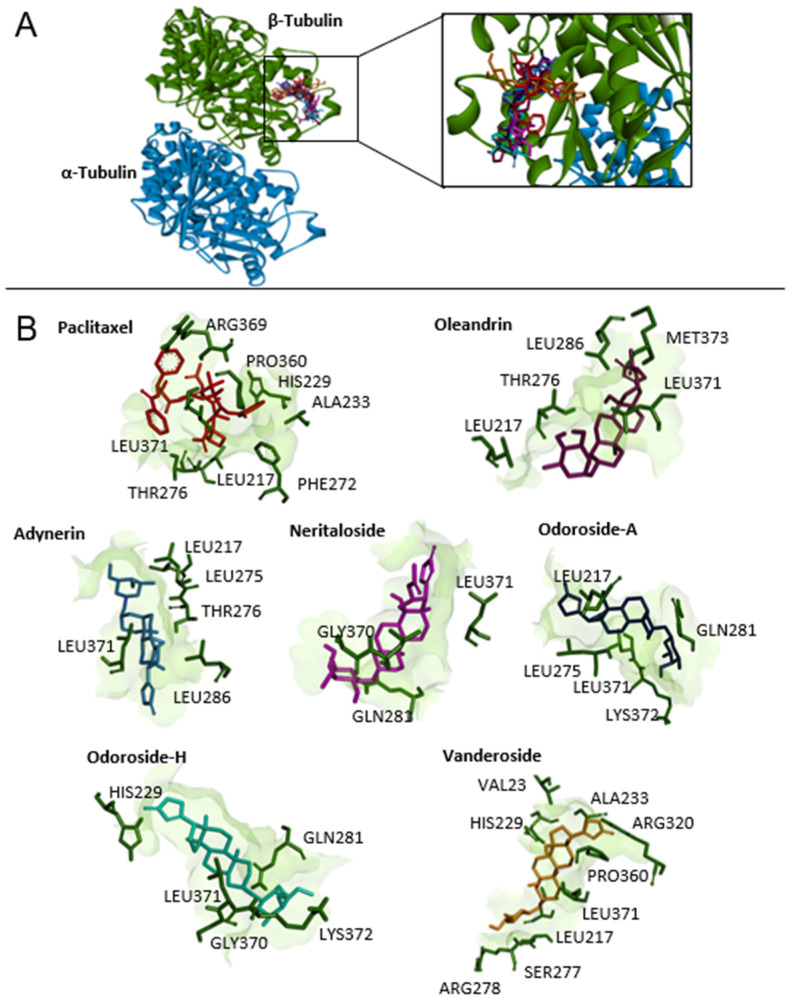
Molecular docking of Breastin’s cardenolides to human α- and β-tubulin using AutoDock 4.2.6. (**A**) The lowest-energy conformation of six compounds and the positive control paclitaxel docked into the taxane-binding pocket of β-tubulin. (**B**) The amino acid residues that are involved in the interaction with β-tubulin were obtained using AutoDockTools and visualized using the Discovery Studio Visualizer software.

**Figure 10:**
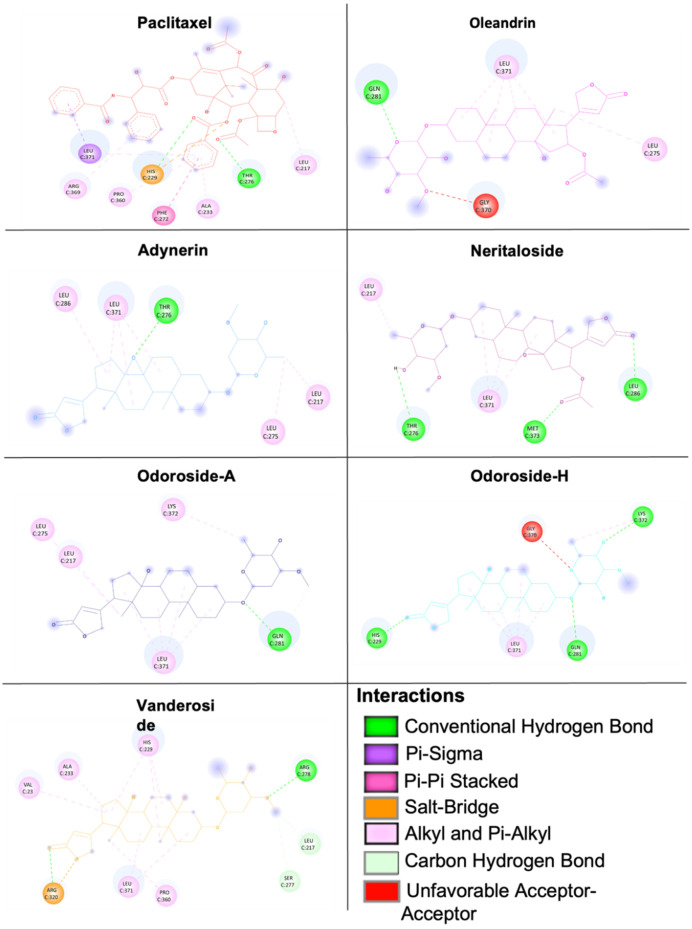
Two-dimensional representation of the binding mode between Breastin’s cardenolides and β-tubulin. Different types of interactions are predicted between the amino acids of β-tubulin in the taxane-binding site and the respective compound as shown by Discovery Studio Visualizer software. Paclitaxel was used as a positive control.

Therefore, we performed defined docking by laying the grid boxes over the main drug-binding sites of tubulin (i.e., the Vinca alkaloid-, taxane-, and colchicine-binding sites). In the defined docking approach, the LBE and predicted inhibition constant (pKi) values were generally lower at the Vinca alkaloid- and taxane-binding sites than at the colchicine-binding site. Vinorelbine and paclitaxel showed the lowest LBE and pKi values at their corresponding binding sites, indicating that the molecular docking approach likely was correct. The results of defined molecular docking revealed that not only odoroside H and neritaloside but also the other cardenolides of Breastin were bound to tubulin, supporting the hypothesis that Breastin inhibits cancer growth, at least in part, by affecting tubulin. The interaction of adynerin, neritaloside, odoroside A, odoroside H, oleandrin, and vanderoside with β-tubulin, illustrated using Discovery Studio Visualizer, is shown in Figure 9. All compounds were found to have the potential to bind to the taxane-binding site but with a different orientation. Figure 10 shows the amino acid residues and their different binding modes with these compounds. Paclitaxel served as a positive control drug.

We used two different docking protocols: Using a blind docking approach, we scanned the whole surface of the target proteins to identify the docking pose of the compounds on the target. These results were subsequently verified by a defined docking approach. Here, we focused only on the specific docking pose identified by blind docking and found consistent results. These two in silico approaches were experimentally verified by two in vitro techniques, i.e., immunofluorescence-based confocal microscopy and a biochemical tubulin binding assay. Therefore, using two in silico and two in vitro assays provided sufficient confidence in the validity of our results.

### 2.9. Proteome Profiling of Odoroside H and Neritaloside

To investigate the influence of protein expression on cellular resistance and sensitivity to odoroside H and neritaloside, we correlated the log_10_IC_50_ values of 59 tumor cell lines with the cellular expression of 3171 proteins as measured by mass spectrometry deposited at the database of the Developmental Therapeutics Program of NCI (USA) (https://dtp.cancer.gov; accessed on 8 February 2023). We selected 40 out of 3171 proteins consisting of the top 20 directly and the top 20 inversely correlating proteins with the log_10_IC_50_ values of these two compounds. The proteins and their biological functions identified for odoroside H are shown in Supplementary Table S1, and those for neritaloside are shown in Supplementary Table S2.

**Figure 11:**
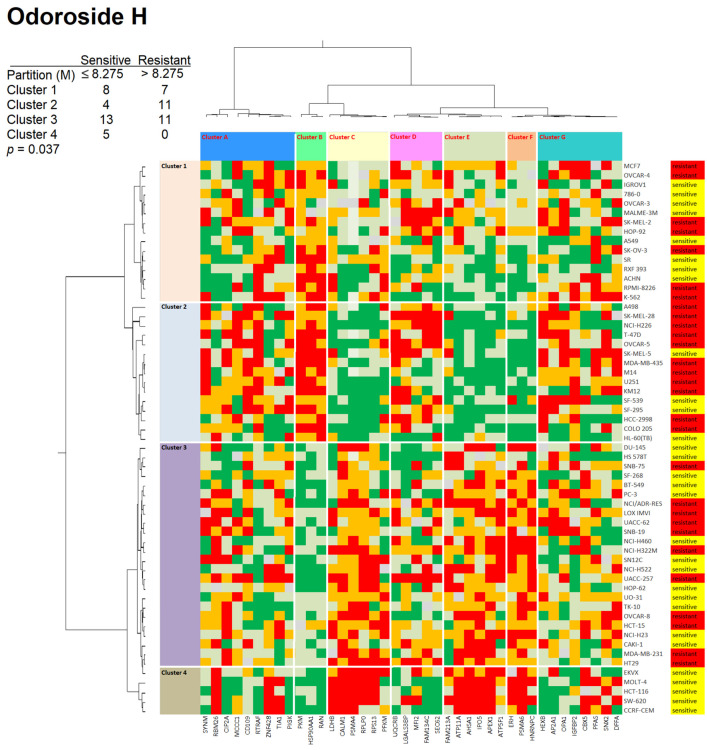
Heat maps and hierarchical cluster analyses of proteins whose expression correlated with the response of 59 tumor cell lines to odoroside H (log_10_IC_50_). The proteins are given in Supplementary Table S1. The cell lines, their tumor origins, and their sensitivity/resistance to odoroside H are shown on the right side of the heat maps. Cell lines with individual log_10_IC_50_ values smaller than the median value of all 59 cell lines tested for odoroside H were defined as sensitive, while all others with log_10_IC_50_ values above the median were defined as resistant. The cell lines were clustered according to their degrees of relatedness to each other on the basis of their protein expression included in the analysis. Color code: red, 0–25% quartile; orange, 26–50% quartile; grey, median value; light green, 50–75% quartile; dark green, 76–100% quartile.

We analyzed the protein expression profiles using hierarchical cluster analysis (Ward method). The cellular expression of these 40 proteins was assembled in the first dimension, and the log_10_IC_50_ values for the two compounds in the second dimension led to two-dimensional color-coded heat maps. For odoroside H, four major clusters were obtained for the 59 tumor cell lines (clusters 1–4), and seven clusters were obtained for the 40 proteins (clusters A–G) (Figure 11). As a next step, we addressed the question of whether the distribution of sensitive and resistant cell lines was statistically different between the cell line clusters. The cell lines were defined as being sensitive or resistant to odoroside H if their log_10_IC_50_ values were smaller or larger than the median values across all 59 cell lines. Indeed, we observed that the distribution of sensitive and resistant cell lines was significantly different (*p* = 0.037, χ^2^).

**Figure 12:**
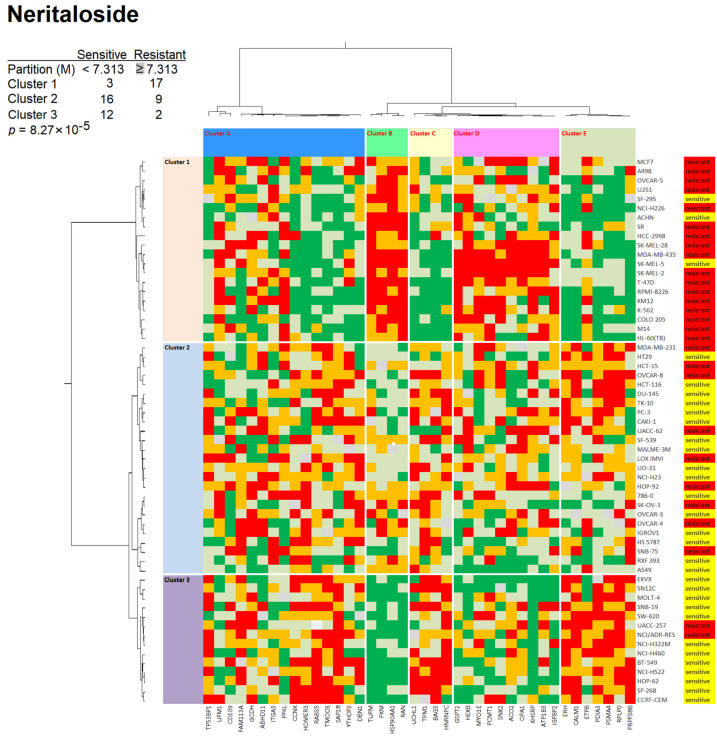
Heat maps and hierarchical cluster analyses of proteins whose expression correlated with the response of 59 tumor cell lines to neritaloside (log_10_IC_50_). The proteins are given in Supplementary Table S2. For details, see Figure 11 legend.

For neritaloside, we found three clusters for the cell lines (clusters 1–3) and another four clusters for the proteins (Figure 12). The distribution of sensitive and resistant cell lines was also statistically different between the cell line clusters (*p* = 8.27 × 10^−5^, χ^2^ test).

The results in Figure 11 and Figure 12 indicate that the protein expression sets were able to predict sensitivity or resistance to odoroside H or neritaloside.

### 2.10. Antitumor Activity of Breastin In Vivo

**Figure 13:**
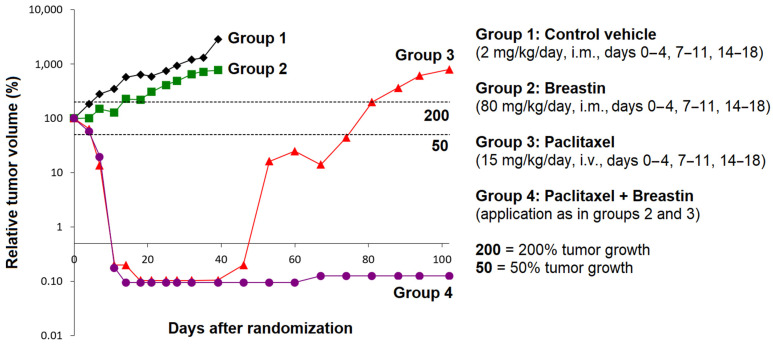
In vivo efficacy of Breastin and paclitaxel in human MAXF 401 xenograft tumor in nude mice.

Finally, we were interested in whether Breastin can inhibit cancer cells not only in vitro but also in animals. For this reason, we used a tumor xenograft model. MAXF 401 cells were transplanted into nude mice. After an initial induction growth phase of MAXF 401 cells for 24 days, the mice were treated with Breastin (80 mg/kg/day, group 2), paclitaxel (15 mg/kg/day, group 3), Breastin plus paclitaxel (same concentrations, group 4), or vehicle control (group 1). The results are shown in Figure 13. On day 0, the tumor size was defined as 100%. Within the following 39 days, the untreated tumors grew from 100% to 2836%, while the Breastin-treated tumors grew only to 770%. Paclitaxel treatment as well as the combination treatment with paclitaxel plus Breastin decreased the tumor size from 100% to 0.1%. After day 39, the paclitaxel-treated tumors became refractory and grew from 0.1% to 801% on day 102. In contrast, the tumors treated with paclitaxel plus Breastin did not become refractory at all.

## 3. Discussion

### 3.1. Phytochemistry

Identification of the constituents by HPLC in the current batch of Breastin revealed the same compounds that have been identified in a previous investigation of another batch, i.e., oleandrin, oleandrigenin sarmentoside, neritaloside, odoroside H, and odoroside A [23]. This measurement was performed for quality control, as it indicates that the chemoprofile of Breastin shows consistency over the years. Another aqueous N. oleander extract (Anvirzel) also contained oleandrin, odoroside, neritaloside, and oleandrigenin [27]. Standardized herbal extracts with constant composition are a prerequisite for high-quality phytotherapy [28]. This is also exemplified by diverse reports in the literature reporting partly different compositions of N. oleander leaf extracts obtained with different solvents [29,30,31]. In addition to the compounds mentioned above, other reported phytochemicals are adynerigenin, odoroside B, β-neriursate neridiginoside, nerizoside, oleandogoside, oleanderocioic acid, oleandiginoside, kaempferol and quercetin glycosides, gallic acid, coumaric acid, jasmonic acid, vanillic acid, ursolic acid, and o-cresol, among others [29,30,31].

### 3.2. Cytotoxicity

Previously, the IC_50_ values of 36 tumor cell lines in a preliminary investigation were reported in cell lines originating from solid tumors but not in hematopoietic tumor cell lines [23]. Inspired by these initial positive results, we analyzed hematopoietic cell lines (leukemia and multiple myeloma cell lines in comparison to cell lines from solid tumors). Thus, the current investigation expands the array of tumor types that could be considered for Breastin treatment. A comparison with cell lines derived from solid tumors showed that the inhibition rates in hematopoietic cell lines were in a similar range or even better. This indicates that Breastin has broad activity in many cancer entities. These results stimulated us to investigate the molecular modes of action in more detail. Breastin has been tested in the cell line panel of Oncotest Ltd., while the two compounds were tested in the NCI Drug Developmental Program. Our intention was not to compare activity cell line by cell line but to show the general susceptibility of different cancer cell lines from different panels to Breastin and the two compounds. We think that showing Breastin’s activity in diverse panels increases the evidence to better demonstrate Breastin’s anticancer activity.

### 3.3. Targets of N. Oleander in Cancer Cells

A COMPARE analysis of the Oncotest cell line panel also indicated that a majority of the top 10 most closely correlated reference anticancer compounds were related to disturbing the mitotic spindle. Therefore, we hypothesized that the mitotic spindle and tubulin, as the most predominant protein in mitosis, might be disturbed by Breastin. Indeed, we were able to verify this assumption by independent in vitro experiments regarding microtubule stabilization (tubulin polymerization assay, confocal microscopy). From the literature, there are no hints that adynerin, odoroside A and H, vanderoside, and neritaloside might inhibit or stabilize microtubules. Oleandrin has been reported to arrest the cell cycle of cancer cells in the G2/M phase, which can be taken as a hint that mitotic arrest might indicate an interaction with microtubules [32]. The ethyl acetate soluble fraction of a methanolic N. oleander leaf extract disrupted the interphase microtubule network and reduced the mitotic index [33]. In general, the correlation coefficients of the COMPARE analysis were rather modest. Nevertheless, it was possible to identify tubulin as a possible target in consecutive, independent experiments, indicating that the data generated with COMPARE analysis were robust enough. 

The isolated compounds were only used to obtain insights into the molecular modes of action of the selected constituents of Breastin. In this sense, the isolated compounds were only tools on our way to explain the activity of Breastin and to bring this extract closer to clinical application. The final goal is to develop Breastin as a herbal preparation for clinical application but not to perform classical drug development with chemical compounds such as odoroside and neritaloside.

Other targets have also been discussed in the literature. Cardenolides (including oleandrin) are well characterized for their inhibitory activity towards Na^+^/K^+^-ATPase [34,35,36,37,38]. N. indicum (which can be considered as a synonym for N. oleander) was described as a Notch inhibitor [39], and N. oleander was found to target fibroblast growth factor 2, Wnt/β-catenin signaling, and the AKT/PI3K/mTOR pathway [40,41,42,43]. N. oleander and oleandrin were found to be inhibitors of the multidrug-resistance-conferring efflux pump P-glycoprotein in most, but not all, investigations [11,44,45,46,47]. Furthermore, oleandrin was found to reduce the transcription factors NF-κB and AP-1 and the c-JUN NH2 terminal kinase [48], increase the phosphorylation of ERK, and decrease the phosphorylation of AKT [33].

The cytotoxic activity of N. oleander is not only due to oleandrin but also due to other compounds in N. oleander such as odoroside A, nerigoside, and oleandrigenin [49,50,51]. Standardized herbal extracts such as Breastin can, therefore, be seen as a combination therapy, since several bioactive molecules simultaneously inhibit cancer cells. Our results on Breastin fit into the general concept that natural products frequently act by more than one mechanism but that addressing multiple targets by natural products does not reflect nonspecificity but multispecificity [52]. In addition, natural products can synergistically enhance and improve the action of anticancer drugs such as paclitaxel [53].

### 3.4. Proteomic Expression Profiling

During the past two decades, it became more and more clear that the sensitivity and resistance of tumor cells to cytotoxic drugs are determined not only by single factors but by transcriptome-wide gene expression (e.g., [54,55,56,57,58]). The generation of cluster image maps (CIMs) or so-called heat maps was pioneered by a consortium of the Harvard Medical School, NCI (Bethesda, MD, USA), and other research institutes [59,60]. In the past years, we investigated this concept for natural products (e.g., [61,62,63]). Rather than transcriptomic mRNA expression, we focused on proteomics in the present investigation. We used a set of previously published proteomic data of 3171 proteins in the NCI panel of tumor cell lines [64] and generated hierarchical cluster analysis-based heat maps to predict the sensitivity or resistance of 59 NCI cell lines to odoroside H and neritaloside. 

The dataset of proteins that were significantly associated with the response of tumor cells to odoroside H usefully complemented our previous mechanism-of-action studies. Several proteins have functions in primary and energy metabolism (PIGK, HEXB, PKM, ATP5F1, TIA1, OPA1), which could be an effect of the Na^+^/K^+^ pump activity. Other proteins are involved in the regulation of proliferation processes (IGFBP2, CIP2A, CD109, CBX5, PSMA4) and may explain the growth-inhibitory activity of odoroside H. Moreover, the expression of several proteins involved in RNA function and processing (RPS13, RPLP0, RAN, RBM26, HNRNPC, ERH) correlated with the anticancer effect of odoroside H. This possibly points to another mechanism of action of odoroside H that is worth investigating further in the future. Interestingly, some potential resistance mechanisms were also found, such as DNA repair (APEX1), chaperone (AHSA1, HSPAA1), and cytoskeletal elements (SYNM).

The cellular response to neritaloside was associated with mechanism classes similar to those of odoroside H, e.g., primary and energy metabolism (HEXB, ABHD11, GCDH, ETFB, PFKL, ACO2, ATP1B3, OPA1, PKM), proliferation (IGFBP2, CD109, SAP18, CCNK, PSMA4), and proteins related to RNA function and processing (PRPF38B, YTHDF3, RPLP0, HNRNPC, RAN, KHSRP, GSPT2, ERH). This indicates that odoroside H and neritaloside may complement each other in their action against cancer cells and possibly enhance each other. The expression of 14 proteins was significantly correlated with sensitivity or resistance to both compounds (IGFBP2, CD109, PSMA4, OPA1, SNX2, HEXB, PKM, FAM213A, RAN, RPLP0, HNRNPC, ERH, CALM1, HSP90AA1). Future experiments should clarify whether combination therapy with both substances has additive or synergistic effects on cancer cells. 

### 3.5. Activity against Xenograft Tumors

The anticancer activity in vivo indicates that Breastin might also be active in human patients. In our experiments, Breastin significantly reduced tumor growth, indicating that Breastin has the potential to prevent both tumor development and progression. Oleandrin indeed inhibited tumor promotion in a murine chemically induced skin carcinogenesis model [60]. For tumor therapy, the clinical situation is rather different from this experimental setting. Upon diagnosis, the tumor has already reached a certain size. Then treatment has to reduce tumor mass. Other authors also observed that oleandrin also reduced tumor growth if the tumor was already established [65].

Furthermore, it was encouraging for us to see that Breastin exerts a strong synergistic effect if combined with the standard anticancer drug paclitaxel. This is a remarkable result. Clearly, Breastin was a dose modifier for paclitaxel. If we assume that Breastin has a mode of action similar to that of paclitaxel (i.e., inhibition of tubulin depolymerization), then a dose intensification of paclitaxel alone should lead to a similar effect. High-dose chemotherapy with paclitaxel is, however, associated with significant toxicities [53,66,67] and might, hence, not be the best treatment choice. The combination with another drug such as Breastin might not increase the same toxicities but scatter the side effects throughout the body to other sites, ultimately leading to better tolerability of the treatment [53].

If Breastin were ever to be established in clinical oncology, then it would probably not be used as monotherapy, but as part of combination regimens. Therefore, Breastin must not generate antagonisms with other drugs but synergisms or at least additive effects. The fact that the relapse of paclitaxel-treated tumors was prevented by the addition of Breastin may be taken as a strong argument for the clinical promise of Breastin. Recently, another study described the synergistic effects of radiotherapy and a supercritical CO_2_ extract of N. oleander in glioblastoma xenograft tumors [68]. Other authors investigated the combination treatment of oleander with anticancer drugs (cisplatin, oxaliplatin, 5-fluorouracil, irinotecan) as well as radiotherapy [69,70,71,72]. All these results can be taken as an indication that the combination of N. oleander extract, or isolated oleandrin, with standard chemotherapy or radiotherapy may exert beneficial effects against tumors.

### 3.6. Acute Toxicity and Side Effects

In general, it is advisable to be careful with cardiac glycosides because they are known for their toxicity. We found some toxic reactions, but they were rather mild and tolerable in our mouse experiments. Another aqueous N. oleander extract (Anvirzel) also only exerted mild toxicities in a clinical phase 1 trial in 18 patients with refractory solid tumors. The side effects included fatigue, nausea, vomiting, dyspnea, and mild injection site pain [73]. A review of cases of oleander intoxication in hospitals revealed that the mortality risk with oleander ingestion was rather low for adults but higher for children [74]. High ingested doses may cause effects on the gastrointestinal system (nausea and vomiting, excess salivation, abdominal pain, diarrhea), heart (arrhythmia, hypotension), and central nervous system (drowsiness, muscle tremors, seizures, collapse, and coma). Although our data with Breastin and those of other authors did not speak for severe and life-threatening side effects, it may be wise to be cautious. Oleander poisoning remains a real threat [5,75,76]. The clinical utility of N. oleander extracts for cancer chemotherapy will ultimately depend on whether it will be possible to have a therapeutic window that is wide enough to effectively kill the tumor but to spare normal tissues with mass treatment strategies with mild and tolerable side effects.

The toxicities of Breastin were already investigated in detail [77]. Briefly, the LD_50_ of Breastin in Albino Balb/C mice was 0.39 mL/25 g i.m. (=15.6 mg/kg), 0.30 mL/25 g i.p., (=12.0 mg/kg), and 0.33 mL/25 g s.c. (=13.2 mg/kg). Tachycardia, myorelaxation, and motoric incoordination were found at high doses, but single or repeated doses i.m., i.p., or s.c. up to 0.15 mL/25 g (6 mL/kg) did not provoke side effects. Animals generally gained weight (all groups), indicating that Breastin was not anorexic at the doses used. Breastin did not decrease blood counts or change hemoglobin contents or blood indices during the treatment applied for 8 weeks in mice. In contrast, Breastin increased the leukocyte counts in the experimental groups. Liver and renal function abnormalities were not observed. Breastin caused neither erythema nor any cutaneous sensitization in the skin of guinea pigs nor any inflammatory changes in the eyes of the tested rabbits as compared to the untreated controls. Given chronically for 6 months in rats, Breastin did not cause significant differences in the kidney profiles but did cause a significant increase in aspartate and alanine aminotransferases. The histopathological studies performed on biopsies obtained from the kidneys and the livers of rats also revealed normal results. However, the microscopic anatomy performed on biopsies taken from the liver of the dead animals showed some changes such as infiltration of inflammatory cells and passive congestion, but no central necrosis was detected. In summary, Breastin did not show severe or life-threatening (grade 3 and 4) toxicities in rodents. However, caution is advised as it is known that rodents have a much higher acceptance (100×) of cardiac glycosides than humans and oleandrin can pass the blood–brain barrier [78,79].

## 4. Material and Methods

### 4.1. Extract Preparation

Breastin is a cold-water extract from leaves of N. oleander. The preparation of the extract has been described [23]. The only difference was the timepoint of the collection of materials. The date of the previous collection was November 2001, when the weather conditions were cold (wintertime in Jordan), whereas the date of collection for the current study was July–August 2019 and the weather conditions were mild (summertime in Jordan). The specimens were deposited at the Royal Botanic Garden and Jordan University (Amman, Jordan). The voucher codes are SN/NC1 and SN/NC2. Nerium oleander (Apocynaceae) leaves were collected from Jordan and were taxonomically identified by direct comparison with authenticated samples of the herbarium of the Biology Department, Faculty of Science, Jordan University, Jordan. Sterile, freshly ground leaves (200 g) of N. oleander were soaked in distilled water (1000 mL) under sterile conditions for at least 8 h. The solution was filtered and the volume was adjusted to 350 mL to obtain a clear, dark brown filtrate. The sterile filtrate was lyophilized under sterile conditions.

### 4.2. Analytical Methods

Proton NMR spectra were recorded using a Varian Unity 500 spectrometer at 499.83 MHz using D_2_O as solvent. Chemical shifts in ppm were referenced to the internal standard TMS (δ = 0). The high-resolution ESI mass spectra were obtained using a Bruker Apex III 70 eV Fourier transform ion cyclotron resonance (FT-ICR) mass spectrometer equipped with a 7.0 Tesla superconducting magnet, an RF-only hexapole ion guide, and an external electrospray ion source. Nitrogen was used as drying gas at 150 °C. The sample solutions were introduced continuously via a syringe pump with a flow rate of 120 µL/h. The data were evaluated using the Bruker XMASS 7.0.8 software.

Low-resolution negative LCMS spectra were obtained from a Finnigan MAT TSQ 7000 instrument (electrospray voltage 4.5 kV; heated capillary temperature 220 °C; sheath gas nitrogen) coupled with a Surveyor MicroLC system equipped with an RP18-column (5 µm, 1 × 100 mm, SEPSERV). An H_2_O:CH_3_CN gradient system containing 0.2% HOAc was used for HPLC measurements.

### 4.3. Cell Lines

The multiple myeloma cell lines KMS11, KMS12BM, NCI-H929, MolP8, JJN3, OPM2, AMO1, and L363 were provided by Dr. Ellen Leich and Manik Chatterjee, University of Würzburg, Würzburg, Germany. RPMI8226 cells were purchased from the American Type Cell Culture Collection (ATCC CCL-155, Manassas, VA, USA). Their maintenance has been described [80].

Human NB4 acute promyelocytic leukemia cells were provided by Dr. Gabriele Greve (Medical Center, University of Freiburg, Breisgau, Germany). Human HL60 acute promyelocytic leukemia cells were provided by Dr. Andreas Schwarting and Dr. Julia Weinmann-Menke (Medical Center, University Mainz, Mainz, Germany). Human MOLT-4 acute lymphoblastic leukemia cells were obtained from the tumor bank of the German Cancer Research Center (Heidelberg, Germany). 

Drug-sensitive CCRF-CEM acute lymphoblastic leukemia cells and P-glycoprotein-overexpressing CEM/ADR5000 cells were cultured as described [81]. The maintenance of the resistance phenotype was accomplished using 5000 ng/mL doxorubicin (provided by the University Medical Center, Mainz, Germany). The multidrug resistance phenotype in CEM/ADR5000 cells has been previously characterized [26,81,82,83]. 

Multiple myeloma and leukemia cell lines were cultured in RPMI 1640 medium supplemented with 10% fetal bovine serum (FBS) (Invitrogen) and 1% penicillin (100 U/mL)–streptomycin (100 µG/mL) (PIS) antibiotic (Invitrogen) and incubated in humidified 5% CO_2_ atmosphere at 37 °C. Cells were passaged twice weekly. All experiments were conducted with the cells in their logarithmic growth phase. 

Seventy-four cell lines were established from patient-derived xenograft tumors (PDX) subcutaneously passaged in nude mice by Oncotest GmbH (Freiburg, Germany). Other cell lines were obtained from ATCC (Rockville, MD, USA), DSMZ (Braunschweig, Germany), or the National Cancer Institute (Bethesda, MA, USA) as described [84,85].

Several other tumor cell lines of the Research Genetic Cancer Centre S.A. (RGCC International GmbH, Zug, Switzerland) have been tested for their sensitivity toward Breastin. We used the following human carcinoma cell lines from human carcinoma types: KRAS-mutated HCT-116 colorectal carcinoma, estrogen-receptor-positive MCF-7 breast carcinoma, triple-negative MDA-MB-231 breast adenocarcinoma, MOR lung adenocarcinoma, PANC-1 ductal cell pancreatic carcinoma, and androgen-responsive LNCaP prostate carcinoma. 

A GFP fusion construct of α-tubulin was used to generate stably transfected U2OS-GFP-α-tubulin cells. The cell line was a generous gift from Joachim Hehl, Light Microscopy Centre, ETH Zürich. Wild-type U2OS cells were obtained from Dr. Wynand Roos (Institute of Toxicology, Medical Center of the University Mainz, Mainz, Germany).

The Developmental Therapeutics Program of the National Cancer Institute (NCI, Bethesda, MD, USA) uses a panel of 55 human tumor cell lines (leukemia, melanoma, brain tumors, and carcinoma of the lung, colon, kidney, ovary, breast, or prostate) for drug screening [86]. The drug screening results (log_10_IC_50_ values obtained by a sulforhodamine 123 assay) and transcriptomic and proteomic expression data were deposited on the NCI website (https://dtp.cancer.gov (accessed on 30 August 2022)).

### 4.4. Cytotoxicity Assays

#### 4.4.1. Resazurin Reduction Assay

The resazurin reduction assay was used to investigate the cytotoxicity of Breastin to tumor cells. The assay is based on the reduction of the indicator dye, resazurin, to the highly fluorescent resorufin by viable but not drug-treated dead cells [87]. Nonviable cells do not exhibit blue staining due to losing their metabolic capacity. The procedure has been described [88,89]. Fluorescence was measured using an Infinite M2000 Proplate reader (Tecan, Crailsheim, Germany) with excitation at 544 nm and emission at 590 nm. Three independent experiments with six replicates each were performed. Dose–response curves of each cell were formed using GraphPad Prism v6.0 software (GraphPad Software Inc., San Diego, CA, USA). The 50% inhibition concentrations (IC_50_) were calculated by nonlinear regression using Microsoft Excel.

#### 4.4.2. Propidium Iodide (PI) Cell Viability Assay

A modified propidium iodide assay [90] was used to assess the compound’s activity in Oncotest’s panel of 74 tumor cell lines. Briefly, cells were harvested from exponential phase cultures by trypsinization, counted, and plated in 96-well flat-bottomed microtiter plates at a cell density depending on the cell line (4000–10,000 cells/well). After a 24 h recovery period to allow the cells to adhere and resume exponential growth, 10 µL of culture medium (six control wells/plate) or culture medium containing the test compounds was added to the cells. The compounds were applied in triplicates at five concentrations. Following four days of continuous drug exposure, the medium or medium with the test compound, including all dead cells suspended in the culture medium, was aspirated and replaced by 200 µL of an aqueous PI solution (7 µg/mL). To measure the fraction of living cells, cells were permeabilized by freezing the plates, resulting in the death of all cells that had remained attached to the bottom of the well after the incubation period. After thawing of the plates, fluorescence was measured using the Cytofluor 4000 micro-plate reader (excitation 530 nm, emission 620 nm), providing a direct relationship with the total viable cell number.

#### 4.4.3. Methyl-Tetrazolium (MTT) Dye Assay

Cell lines of the RGCC panel were tested with the MTT assay as described [91] with a concentration range of 0.01, 0.5, 1, 2.5, 5, 10, and 50 µg/mL Breastin. 4-Hydroxycyclophosphamide (CAS:40277-05-2; Cat. No. 7228CA, AK Scientific, Inc., Union City, CA, USA) was used as a control drug. The experiments of cellular proliferation determination were performed in triplicates. The average absorbance was calculated for each triplicate. Subsequently, the sample measurements were corrected for the measurement of the blank. The differences in the mean were determined with the one-sample t-test by comparing the treated samples with the untreated controls. A statistically significant difference was considered for *p* values < 0.05. Results were calculated using Microsoft Excel 2016. The measurements from the proliferation assays were used to determine the IC_50_ values upon 48 h incubation by using Microsoft Excel 2016.

### 4.5. Imaging of Structure and Dynamics of the Microtubule Cytoskeleton by Fluorescence Microscopy

The method has been described [76]. Aliquots of 2 × 10^4^ U2OS-GFP-α-tubulin cells were seeded in each well of a sterile ibiTreat μ-slide (Ibidi, Germany), and cells were allowed to attach overnight. Cells were treated with 13.2 μg/mL Breastin or DMSO (solvent control) and incubated at 37 °C for 2 h. After rinsing with PBS and staining for 15 min with 300 nM 4′,6-diamidino-2-phenylindole (DAPI) (Life Technologies, Darmstadt, Germany), the cells were washed with PBS and mounted. Fluorescence imaging was performed by using 470 nm excitation and 525 nm emission for GFP and 360 nm excitation and 447 nm emission for DAPI with an EVOS digital inverted microscope (Life Technologies). Each experiment was repeated at least three times and representative images were selected.

### 4.6. Tubulin Polymerization Assay

Breastin was investigated by using the In Vitro Tubulin Polymerization Assay Kit (Merck, Darmstadt, Germany) following the manufacturer’s instructions. The analyses were accomplished using a FlexStation 3 Multi-Mode Microplate Reader (Molecular Devices, San Jose, CA, USA). The readings were obtained by measuring the turbidity variation (light scattering) every 30 s for 3 h (360 measurements in total) at 350 nm [80].

### 4.7. COMPARE Analysis

The COMPARE algorithm [92,93] was applied to the analysis of the growth inhibition data of Breastin and its constituents to obtain clues regarding its possible mechanism of action.

The log_10_C_50_ values for odoroside H and neritaloside were deposited in the database of the NCI, USA (https://dtp.cancer.gov; accessed on 30 August 2022) and correlated with the corresponding log_10_C_50_ values of 91 standard anticancer agents determined against the NCI tumor cell line panel. These standard agents represent the main molecular mechanisms of action for established anticancer drugs. Similarities between the sensitivity pattern of a test compound and the sensitivity pattern of standard drugs were expressed quantitatively as Pearson correlation coefficients. High-level correlations between the sensitivity patterns of two compounds are strongly suggestive of a similar mode of action.

As a second independent approach, we performed *COMPARE* analyses with the cell line panel of Oncotest GmbH (Freiburg, Germany). Here, the IC_50_ and IC_70_ values of Breastin were correlated with the corresponding IC_50_/IC_70_ values of 153 standard agents with known modes of action determined against the same cell line panel. These standard agents represent the main molecular mechanisms of action for established anticancer drugs. Similarities between the sensitivity pattern of a test compound and the sensitivity pattern of standard drugs are expressed quantitatively as Spearman correlation coefficients [94]. High-level correlations between the sensitivity patterns of two compounds are strongly suggestive of a similar mode of action.

### 4.8. Molecular Docking

The three-dimensional structures of six phytochemicals from Breastin (adynerin, neritaloside, oleandrin, vanderoside, odoroside A, and odoroside H) were downloaded from PubChem (http://www.PubChem.nih.gov; accessed on 30 August 2022). The crystal structure of tubulin (PDB ID: 5N5N) was obtained from the Protein Data Bank (http://www.rcsb.org/; accessed on 30 August 2022). AutoDockTools 1.5.6 was used to convert all six compounds, tubulin microtubule inhibitors (vinorelbine, paclitaxel, and colchicine), and tubulin to Protein Data Bank Partial Charge and Atom Type (PDBQT) format. Four different grid boxes were prepared around the whole tubulin protein, the Vinca alkaloid-binding site, the taxol-binding site, and the colchicine-binding site to study the tubulin-binding behavior of Breastin compounds in silico. In AutoDock4.2.6. software, Lamarckian Algorithm was set with 10 runs and 2,500,000 energy evaluations to generate the docking log file. Binding energy and predicted Ki were collected from the RMSD cluster analysis while the interacting amino acids were identified with AutoDockTools [95]. Discovery Studio Visualizer software was used to create the visualizations of protein–ligand interactions.

### 4.9. Hierarchical Cluster Analyses of Proteomic Expression Data

The Pearson correlation test and hierarchical cluster analyses were performed by using mass-spectrometry-based proteomic expression data of 59 NCI cell lines (https://dtp.cancer.gov; accessed on 30 August 2022) [64] to generate a rank-ordered list consisting of the top 20 proteins that directly and the top 20 proteins that inversely correlated with the resistance odoroside H and neritaloside based on the log_10_IC_50_ values of the cell lines. Heat maps based on agglomerative hierarchical clustering according to the Ward method were generated by using the CIM miner software (https://discover.nci.nih.gov/cimminer/oneMatrix.do; accessed on 30 August 2022) [96]. The linear dependency between responsiveness to odoroside H and neritaloside and the expression of 40 selected proteins in the NCI cell line panel was calculated by using the χ^2^ (WinStat, Kalmia, CA, USA).

### 4.10. In Vivo Experiments with Human Xenograft Tumors

In-house outbred NMRI nude mice were used (20–22 g, 4–6 weeks of age). The animals were maintained in Macrolon cages under natural daylight cycles at an ambient temperature of 24 ± 1 °C. Mice were fed with standard chow (Altromin, Lage) and water ad libitum. All animal experiments were approved by the Regierungspräsidium Freiburg, and studies were conducted following the UKCCR guidelines for the use of animals in experimental neoplasia [97].

To study the antitumor efficacy of Breastin in human MAXF 401 xenograft breast cancer, mice were subcutaneously injected with 2 × 10^7^ tumor cells resuspended in 100% Matrigel. The antitumor activity was monitored for Breastin or paclitaxel monotherapy and the combination of Breastin with paclitaxel. Mice were randomized into four treatment groups (6–7 animals per group) if tumors reached 50–250 mm^3^ (control, Breastin, paclitaxel, Breastin plus paclitaxel). The drugs were administered intraperitoneally at 40 mg/kg of BW three times a week for a duration of three to four weeks. Tumor size was measured twice weekly via calipers. Antitumor activity was evaluated as maximum tumor volume inhibition compared with the vehicle control group (optimal T/C values calculated based on median values). Before euthanasia, tumors were carefully excised and immediately snap-frozen in liquid nitrogen for further analysis.

## 5. Conclusions

Breastin inhibits cancer in vitro and in vivo in hematopoietic cell lines (multiple myeloma, leukemia) and epidermal cell lines (diverse carcinomas). Phytochemical profiling revealed the monoglycosidic cardenolides adynerin, neritaloside, odoroside A, odoroside H, oleandrin, and vanderoside as major bioactive secondary metabolites of Breastin. Major molecular mechanisms of drug resistance were not correlated with the activity of odoroside H and neritaloside. In silico and in vitro mode-of-action analyses revealed that Breastin is a mitotic spindle poison that inhibits the depolymerization of microtubules. The inhibition of human xenograft tumors and the observed prevention of tumor relapse by Breastin after treatment with paclitaxel suggest Breastin as a promising cotreatment. These findings may justify clinical investigations in human cancer patients in the future. Special attention has to be paid to the dosing and toxicity profile in patients.

## Figures and Tables

**Table 1:** FTICR-HRMS data of major peaks (see Figure 2).

*m*/*z*	Peak Intensity	Formula	Error (ppm)	Ion	Annotation
**Positive mode:**					
219.02586	1.64 × 10^5^	C_6_H_12_O_6_K^+^	3.1	[M+K]^+^	glucose
247.05716	7.30 × 10^5^	C_11_H_12_O_5_Na^+^	2.2		unknown
365.10317 381.07820 723.19407	2.39 × 10^5^ 1.12 × 10^6^ 2.90 × 10^5^	C_12_H_22_O_11_Na^+^ C_12_H_22_O_11_K^+^ C_22_H_44_O_22_K^+^	6.2 3.1 2.1	[M+Na]^+^ [M+K]^+^ [2M+K]^+^	sucrose
555.26986	1.01 × 10^6^	C_30_H_44_O_7_K^+^	3.6	[M+K]^+^	3-O-diginosyl-8,14-epoxy-3-hydroxycard-20(22)-enolide (adynerin), anhydrodigitoxigenin sarmentoside or -diginoside
557.28570	8.37 × 10^5^	C_30_H_46_O_7_K^+^	3.3	[M+K]^+^	digitoxigenindiginoside (odoroside A)
571.26625	7.30 × 10^5^	C_30_H_44_O_8_K^+^	0.9	[M+K]^+^	anhydrodigitoxigenindigitaloside, 3-O-digitalosyl-8,14-epoxy-3-hydroxycard-20(22)-enolide
573.28129	1.31 × 10^6^	C_30_H_46_O_8_K^+^	1.9	[M+K]^+^	digitoxigenindigitaloside (odoroside H), 3-O-diginosyl-3,5,14-trihydroxycard-20(22)-enolide (vanderoside)
599.31794 615.29285	2.15 × 10^5^ 1.74 × 10^6^	C_32_H_48_O_9_Na^+^ C_32_H_48_O_9_K^+^	1.9 0.2	[M+Na]^+^ [M+K]^+^	oleandrigenin-oleandroside (oleandrin), -sarmentoside or -diginoside
631.28746	5.48 × 10^5^	C_32_H_48_O_10_K^+^	0.7	[M+K]^+^	oleandrigenindigitaloside (neritaloside)
**Negative mode:**					
191.05760	3.60 × 10^6^	C_7_H_11_O_6_^−^	7.8	[M−H]^−^	quinic acid
353.09100	5.23 × 10^6^	C_16_H_17_O_9_^−^	9.0	[M−H]^−^	chlorogenic acid
341.11213 377.08913 719.21095	1.76 × 10^6^ 5.35 × 10^6^ 1.71 × 10^6^	C_12_H_21_O_11_^−^ C_12_H_22_O_11_Cl^−^ C_24_H_44_O_22_Cl^−^	9.4 9.3	[M−H]^−^ [M+Cl]^−^ [2M+Cl]^−^	sucrose
609.15238	2.51 × 10^6^	C_27_H_29_O_16_l^−^	10.3	[M−H]^−^	rutin

**Table 2:** Correlation of log_10_IC_50_ values for odoroside H and neritaloside with ABC-transporter mediated mechanisms of multidrug resistance (P-glycoprotein/ABCB1, ABCB5, ABCC1, and ABCG2) and other mechanisms of anticancer drug resistance (EGFR, RAS, TP53, WT1, HSP90, GSTπ, and the proliferation rate of the tumor cell lines).

		Odoroside H	Neritaloside	Control Drug
		(log_10_IC_50_, M)	(log_10_IC_50_, M)	(log_10_IC_50_, M)
**ABCB1 Expression**				Epirubicin
7q21 (Chromosomal	*r*-value	−0.039	0.004	* 0.447
Locus of *ABCB1* Gene)	*p*-value	0.390	0.489	* 3.55 × 10^−4^
*ABCB1* Expression	*r*-value	−0.051	−0.022	* 0.533
(Microarray)	*p*-value	0.350	0.436	* 6.82 × 10^−6^
*ABCB1* Expression	*r*-value	−0.036	−0.104	* 0.410
(RT-PCR)	*p*-value	0.401	0.233	* 1.54 × 10^−3^
Rhodamine 123	*r*-value	−0.023	0.022	* 0.526
Accumulation	*p*-value	0.433	0.435	* 1.12 × 10^−5^
**ABCB5 Expression**				Maytansine
*ABCB5* Expression	*r*-value	0.107	0.140	* 0.454
(Microarray)	*p*-value	0.209	0.143	* 6.67 × 10^−4^
*ABCB5* Expression	*r*-value	0.140	0.227	* 0.402
(RT-PCR)	*p*-value	0.143	* 0.040	* 0.0026
**ABCC1 Expression**				Vinblastine
DNA Gene	*r*-value	0.008	0.010	* 0.429
Copy Number	*p*-value	0.477	0.233	* 0.001
*ABCC1* Expression	*r*-value	−0.141	−0.254	* 0.398
(Microarray)	*p*-value	0.145	* 0.027	* 0.003
*ABCC1* Expression	*r*-value	−0.151	−0.265	0.299
(RT-PCR)	*p*-value	0.156	0.0357	* 0.036
**ABCG2 Expression**				Pancratistatin
*ABCG2* Expression	*r*-value	−0.099	−0.150	* 0.329
(Microarray)	*p*-value	0.230	0.131	* 0.006
ABCG2 Expression	*r*-value	−0.049	−0.058	* 0.346
(Western Blot)	*p*-value	0.356	0.332	* 0.004
**EGFR Expression**				Erlotinib
*EGFR* Gene	*r*-value	0.013	−0.194	−0.245
Copy Number	*p*-value	0.459	0.069	* 0.029
*EGFR* Expression	*r*-value	0.090	−0.275	* −0.458
(Microarray)	*p*-value	0.248	* 0.017	* 1.15 × 10^−4^
*EGFR* Expression	*r*-value	0.143	−0.111	* −0.379
(PCR Slot Blot)	*p*-value	0.143	0.203	* 0.002
EGFR Expression	*r*-value	−0.052	−0.293	* −0.376
(Protein Array)	*p*-value	0.349	* 0.012	* 0.001
***N-/K-/H-RAS* Mutations**				Melphalan
*TP53* Mutation	*r*-value	0.131	0.182	* 0.367
(cDNA Sequencing)	*p*-value	0.161	0.084	* 0002
**TP53 Mutation**				5-Fluorouracil
*TP53* Mutation	*r*-value	−0.222	−0.218	* −0.502
(cDNA Sequencing)	*p*-value	0.047	0.050	* 3.50 × 10^−5^
TP53 Function	*r*-value	−0.119	0.115	* −0.436
(Yeast Functional Assay)	*p*-value	0.195	0.204	* 5.49 × 10^−4^
**WT1 Expression**				Ifosfamide
WT1 Expression	*r*-value	−0.019	0.010	* −0.316
(Microarray)	*p*-value	0.442	0.469	* 0.007
**GSTP1 Expression**				Etoposide
*GSTP1* Expression	*r*-value	−0.124	−0.071	**0.399**
(Microarray)	*p*-value	−0.0173	0.296	* 9.58 × 10^−4^
*GST* Expression	*r*-value	0.008	−0.064	**0.509**
(Northern Blot)	*p*-value	0.474	0.315	* 2.24 × 10^−5^
**HSP90 Expression**				Geldanamycin
*HSP90* Expression	*r*-value	−0.011	0.466	* −0.392
(Microarray)	*p*-value	0.076	0.283	* 0.001
**Proliferation**				5-Fluorouracil
Cell Doubling	*r*-value	0.079	−0.008	* 0.627
	*p*-value	0.279	0.477	* 7–14 × 10^−6^

* *r* > 0.3 or <−0.3 and *p* < 0.05.

**Table 3:** COMPARE analysis of Breastin vs. 153 anticancer agents with known mechanisms in the Oncotest panel of 74 tumor cell lines.

No.	*R*-Value	*p*-Value	Standard Agent	Mode of Action	Mode of Action
**IC_50_-Based Spearman Rank Correlation**					
1	0.576	0.0014	VER-49009	Heat shock protein 90 inhibitor	
2	0.492	0.0081	4-Hydroperoxy-ifosphamide	Alkylating agent	
3	0.429	0.0230	GSK461364A	PLK1 inhibitor	Mitosis-related
4	0.390	0.0403	MST-312	Telomerase inhibitor	
5	0.381	0.0453	BI2536	PLK1 inhibitor	Mitosis-related
6	0.378	0.0470	Thiotepa	Alkylating agent	
7	0.376	0.0488	BI 6727 3 HCl	PLK1 inhibitor	Mitosis-related
**IC_70_-Based Spearman Rank Correlation**					
1	0.487	0.0087	Vinorelbine bistartrate	Tubulin inhibitor	Mitosis-related
2	0.470	0.0112	4-Hydroperoxy-ifosphamide	Alkylating agent	
3	0.455	0.0151	VER-49009	Heat shock protein 90 inhibitor	
4	0.446	0.0176	Ispinesib, mesylate	Eg5 inhibitor	Mitosis-related
5	0.428	0.0230	Methotrexate hydrate	Antimetabolite	
6	0.424	0.0246	Vindesine sulfate	Tubulin inhibitor	Mitosis-related
7	0.415	0.0282	BI6727 3HCl	PLK1 inhibitor	Mitosis-related
8	0.404	0.0333	BI2536	PLK1 inhibitor	Mitosis-related
9	0.395	0.0374	Vincristine sulfate	Tubulin inhibitor	Mitosis-related
10	0.395	0.0377	Vinflunine di-tartrate	Tubulin inhibitor	Mitosis-related
11	0.391	0.0394	Purvalanol A	CDK inhibitor	
12	0.389	0.0409	Suberic bis-hydroxamic acid	HDAC inhibitor	
13	0.380	0.0463	Bleomycin sulfate	DNA synthesis inhibitor	
14	0.380	0.0460	GSK461364A	PLK1 inhibitor	Mitosis-related

**Table 4:** Molecular docking (blind and defined modes) of Breastin constituents to the *Vinca* alkaloid, taxane-, and colchicine-binding sites of β-tubulin. Shown are the lowest binding energies (LBEs, kcal/mol), predicted inhibition constants (pKi, µM), and amino acids involved in ligand interaction with β-tubulin. Vinorelbine, paclitaxel, colchicine, and nocodazole served as control compounds.

Tubulin Binding Sites	Compounds	LBE (kcal/mol)	pKi (µM)	Amino Acids Involved in Ligand Interaction
Blind docking	Vinorelbine	−10.28 ± 0.46	0.04 ± 0.03	HIS197, SER198, ASP199, VAL260, PRO263
	Paclitaxel	−9.45 ± 0.08	0.12 ± 0.02	GLN256, THR257, VAL260, PRO261, ALA314, CYS347
	Colchicine	−6.82 ± 1.01	22.58 ± 15.61	ASP199, PRO263, HIS406, TRP407
	Nocodazole	−6.31 ± 0.11	23.65 ± 4.43	THR382, ALA385, ALA426, GLU429, GLU433
	Adynerin	−8.52 ± 0.16	0.59 ± 0.16	CYS12, THR145, VAL171, SER174, GLU183, ASP205, ASN206
	Neritaloside	−8.80 ± 0.28	0.40 ± 0.19	LYS163, LEU195, GLN256, VAL260, PRO263, HIS266
	Oleandrin	−8.42 ± 0.04	0.67 ± 0.04	LYS163, GLU196, ASP199, THR257, VAL260, PRO263, HIS266
	Vanderoside	−8.66 ± 0.02	0.45 ± 0.01	GLN11, CYS12, GLN15, SER140, VAL171, SER174, GLU183, ASP205, ASN206, TYR 224
	Odoroside A	−8.15 ± 0.02	1.06 ± 0.04	VAL23, LEU217, HIS229, ALA233, SER236, THR276, ARG320, PRO360, LEU371
	Odoroside H	−8.06 ± 0.18	1.29 ± 0.36	GLN11, CYS12, GLU71, ASP205
*Vinca* alkaloid-binding site (defined docking)	Vinorelbine	−10.86 ± 0.39	0.01 ± 0.00	CYS12, SER140, LEU141, GLY142, VAL171, SER174, VAL177, SER178, ASP179, GLU183, ILE204, ASN206, TYR224
	Adynerin	−8.91 ± 0.15	0.37 ± 0.09	CYS12, ALA99, SER140, VAL171, SER178, ASP179
	Neritaloside	−9.75 ± 0.23	0.05 ± 0.03	GLN11, CYS12, PRO173, SER174, GLU183, ASN206, TYR210, TYR224
	Oleandrin	−8.73 ± 0.53	1.10 ± 0.79	LYS176, VAL177, TYR210, ASP211, PHE214, PRO222, TYR224
	Vanderoside	−9.87 ± <0.01	0.04 ± 0.00	CYS12, GLN15, SER140, VAL171, PRO173, SER174, GLU183, ASP205, ASN206, TYR224, GLY225
	Odoroside A	−9.15 ± 0.05	0.17 ± 0.02	CYS12, ALA99, ASN101, THR145, VAL171, SER174, ASN206, GLU207
	Odoroside H	−9.08 ± 0.04	0.29 ± 0.02	CYS12, GLU71, ASN101, THR145, VAL171, SER174
Taxane-binding site (defined docking)	Paclitaxel	−9.93 ± 0.29	0.14 ± 0.11	LEU217, HIS229, ALA233, PHE272, THR276, PRO360, ARG369, LEU371
	Adynerin	−8.02 ± 0.03	1.93 ± 0.03	LEU217, LEU275, THR276, LEU286, LEU371
	Neritaloside	−8.19 ± 0.11	1.02 ± 0.17	LEU275, GLN281, GLY370, LEU371
	Oleandrin	−8.50 ± 0.21	0.63 ± 0.21	LEU217, THR276, LEU286, LEU 371, MET373
	Vanderoside	−7.92 ± 0.07	1.61 ± 0.19	VAL23, LEU217, HIS229, ALA233, SER277, ARG278, ARG320, PRO360, LEU371
	Odoroside A	−8.14 ± 0.08	2.91 ± 0.69	LEU217, LEU275, GLN281, LEU371, LYS372
	Odoroside H	−8.36 ± 0.11	1.47 ± 0.84	HIS229, GLN281, GLY370, LEU371, LYS372
Colchicine-binding site (blind docking)	Colchicine	−7.57 ± 0.01	2.83 ± 0.04	ASP69, THR145, ALA180, TRY224, LEU248, LYS254
	Adynerin	−6.77 ± 0.05	10.91 ± 0.91	GLY10, ALA99, SER178, ALA180, LEU248, LYS254
	Neritaloside	−5.80 ± 0.14	57.77 ± 13.31	LEU70, GLU71, ALA99, VAL177, THR179, ALA180, GLU183, ASN206, TYR224, LEU248
	Oleandrin	−5.26 ± 0.14	144.33 ± 31.57	ALA12, GLU71, ALA99, PRO173, VAL177, ALA180, GLU183, ASN206, TYR210, TYR224, LEU248, LYS254, ASP329
	Vanderoside	−5.51 ± 0.22	98.22 ± 33.98	GLN11, ASP69, LEU70, GLU71, VAL74, ASP98, TYR210, LEU248, LYS254, ASP329
	Odoroside A	−8.19 ± 0.12	1.02 ± 0.20	GLN11, LEU70, GLU71, ALA99, THR145, ALA180, GLU183, TYR224, LEU248
	Odoroside H	−6.68 ± 0.08	12.90 ± 1.60	GLN11, LEU70, GLU71, ALA99, THR179, ALA180, TYR210, TYR224, LEU248, LYS254, ASP329

## Data Availability

Not applicable.

## Supplementary Materials

The following supporting information can be downloaded at: https://www.mdpi.com/article/10.3390/molecules28041871/s1, Table S1: Pearson correlation test-based COMPARE analysis of proteins directly or inversely correlating with the log_10_IC_50_ values for odoroside H in 59 tumor cell lines. The protein functions have been extracted from the GeneCards database (https://www.genecards.org; accessed on 30 August 2022); Table S2: Pearson correlation test-based COMPARE analysis of proteins directly or inversely correlating with the log_10_IC_50_ values for neritaloside H in 59 tumor cell lines. The protein functions have been extracted from the GeneCards database (https://www.genecards.org; accessed on 30 August 2022).

## Sample Availability

Samples of the compounds described in this manuscript are available from the authors.
