# Supplementary material for: Molecular Modes of Action of an Aqueous Nerium oleander Extract in Cancer Cells In Vitro and In Vivo
Source: Molecules. 2023 Feb 16;28(4):1871. doi: 10.3390/molecules28041871 (PMC9960564; doi:10.3390/molecules28041871)
Supplement: Supplementary file 1 [file molecules-28-01871-s001.zip › molecules-1919010-supplementary.pdf]

Supplementary Table S1. Pearson correlation test-based COMPARE analysis of proteins directly or inversely correlating with the $\log_{10}IC_{50}$ values for odoroside H in 59 tumor cell lines. The protein functions have been extracted from the GeneCards database (https://www.genecards.org; accessed on 30 August 2022).

Symbol	*r*-Value	*p*-Value	Entrez Gene ID	Name	Function
PCMT1	0.435	2.83×10^{-4}	5110	Protein-L-isoaspartate (D-aspartate) O-methyltransferase	Type II class of protein carboxyl methyltransferase enzymes. Protein repair by recognizing and converting D-aspartyl and L-isoaspartyl residues resulting from spontaneous deamidation back to the normal L-aspartyl form
HSP90AA1	0.421	4.53×10^{-4}	3320	Heat shock protein 90 α family class A member 1	Molecular chaperone
IGFBP2	0.419	4.85×10^{-4}	3485	Insulin-like growth factor-binding protein 2	Binds insulin-like growth factors I and II
PFKL	0.410	6.31×10^{-4}	5211	Phosphofructokinase, liver type	Catalyzes the conversion of D-fructose 6-phosphate to D-fructose 1,6-bisphosphate, which is a key step in glycolysis
ATP1B3	0.404	7.51×10^{-4}	483	ATPase Na^+/K^+ transporting subunit β 3	Integral membrane protein maintaining the electrochemical gradients of Na and K ions across the plasma membrane
MYO1E	0.403	7.69×10^{-4}	4643	Myosin IE	Nonmuscle class I myosin acting as actin-based molecular motors
CD109	0.400	8.34×10^{-4}	135228	150 KDa TGF-β-1-binding protein	Regulates negatively the signalling by transforming growth factor β
SNX2	0.398	8.89×10^{-4}	6643	Sorting nexin 2	Protein sorting in the endocytic pathway
ETFB	0.398	9.02×10^{-4}	2109	Electron transfer flavoprotein subunit β	Electron-transfer-flavoprotein involved in mitochondrial fatty acid and amino acid catabolism
UFM1	0.396	9.52×10^{-4}	51569	Ubiquitin fold modifier 1	Ubiquitylation of target proteins
GSPT2	0.391	0.001	23708	G1 to S phase transition 2	GTP-binding elongation factor that mediates translation termination and mRNA stability
ABHD11	0.384	0.001	83451	Abhydrolase domain containing 11	*hydrolase activity, acting on ester bonds*
RAN	0.383	0.001	9406	Ras-related nuclear protein	Small GTP binding protein of the RAS superfamily that is essential for the translocation of RNA and proteins through the nuclear pore complex. The RAN protein is also involved in control of DNA synthesis and cell cycle progression
GCDH	0.379	0.002	2639	Glutaryl-CoA dehydrogenase	Catalyzes the oxidative decarboxylation of glutaryl-CoA to crotonyl-CoA and CO(2) in the degradative pathway of L-lysine, L-hydroxylysine, and L-tryptophan metabolism
TUFM	0.368	0.002	7284	Tu translation elongation factor, mitochondrial	Participates in protein translation in mitochondria
ACO2	0.368	0.002	50	Aconitase 2	Catalyzes the interconversion of citrate to isocitrate via cis-aconitate in the second step of the TCA cycle
KHSRP	0.365	0.002	8570	KH-type splicing regulatory protein	Multifunctional RNA-binding protein implicated in a variety of cellular processes, including transcription, alternative pre-mRNA splicing, and mRNA localization
OPA1	0.362	0.002	4976	OPA1 mitochondrial dynamin-like GTPase	Regulation of mitochondrial stability and energy output. This protein sequesters cytochrome c
PKM	0.362	0.002	5315	Pyruvate kinase M1/2	Catalyzes the transfer of a phosphoryl group from phosphoenolpyruvate to ADP, generating ATP and pyruvate
HEXB	0.361	0.003	3074	Hexosaminidase subunit β	Catalyzes the degradation of the ganglioside GM2 and other molecules containing terminal N-acetyl hexosamines
TMOD3	-0.449	1.84×10^{-4}	29766	Tropomodulin 3	Cadherin-binding activity involved in cell-cell adhesion; Involved in actin filament organization
CCNK	-0.441	2.33×10^{-4}	8812	Cyclin K	Regulates transcription through regulating CDK and RNA polymerase II activities
TPM1	-0.413	5.76×10^{-4}	7168	Tropomyosin α-1 chain	Actin-binding protein

TP53BP1	-0.406	7.07×10^{-4}	7158	Tumor protein p53-binding protein 1	DNA double-strand break repair though promoting non-homologous end joining (NHEJ); Promoting checkpoint signaling following DNA damage acting as a scaffold for recruitment of DNA damage response proteins to damaged chromatin
PSMA4	-0.393	0.001	5685	Proteasome 20S subunit α 4	Degradation of peptides and proteins; Involved in mitotic G1 and G1/S phase transition
DBN1	-0.389	0.001	1627	Drebrin 1	Cytoplasmic actin-binding protein involved in neuronal growth
FAM213A	-0.385	0.001	84293	Peroxiredoxin-like 2A	Antioxidant activity
CALM1	-0.385	0.001	801	Calmodulin 1	Calcium-binding protein. Calcium-induced activation of calmodulin regulates and modulates the function of cardiac ion channels
BAG5	-0.378	0.002	9529	BAG cochaperone 5	Anti-apoptotic protein that interactions with cell apoptosis and growth related proteins
SAP18	-0.376	0.002	10284	Sin3A-associated protein 18	component of the histone deacetylase complex.interacts with SIN3 and enhances SIN3-mediated transcriptional repression
UCHL1	-0.375	0.002	7345	Ubiquitin C-terminal hydrolase L1	Thiol protease hydrolyzing peptide bonds at the C-terminal glycine of ubiquitin
HOMER3	-0.369	0.002	9454	Homer scaffold protein 3	Postsynaptic density scaffolding protein that mediates protein-protein interactions
RPLP0	-0.368	0.002	6175	Ribosomal protein lateral stalk subunit P0	Component of the 60S subunit
HNRNPC	-0.366	0.002	3183	Heterogeneous nuclear ribonucleoprotein C	Pre-mRNA processing and other aspects of mRNA metabolism and transport
PDIA3	-0.362	0.002	2923	Protein disulfide isomerase family A member 3	protein of the endoplasmic reticulum that interacts with lectin chaperones calreticulin and calnexin to modulate folding of newly synthesized glycoproteins
RAB35	-0.359	0.003	11021	RAB35, member RAS oncogene family	GTPase activity, involved in endosomal transport
PRPF38B	-0.356	0.003	55119	Pre-mRNA processing factor 38B	Involved in mRNA splicing via spliceosome
YTHDF3	-0.356	0.003	253943	YTH N6-methyladenosine RNA-binding protein 3	RNA-binding
ITGA5	-0.354	0.003	3678	Integrin subunit α 5	Cell surface adhesion and signaling, may promote tumor invasion
ERH	-0.351	0.003	2079	ERH mRNA splicing and mitosis actor	Methyl-CpG binding activity; involved in pyrimidine nucleoside metabolic processes

Supplementary Table S2. Pearson correlation test-based COMPARE analysis of proteins directly or inversely correlating with the $log_{10}IC_{50}$ values for neritaloside H in 59 tumor cell lines. The protein functions have been extracted from the GeneCards database (https://www.genecards.org; accessed on 30 August 2022).

Symbol	*r*-Value	*p*-Value	Entrez Gene ID	Name	Function
FAM134C	0.409	6.57×10^{-4}	162427	Reticulophagy regulator family member 3	Involved in positive regulation of neuron projection development
HSP90AA1	0.397	9.39×10^{-4}	3320	Heat shock protein 90 α family class A member 1	Chaperone function
MCCC1	0.395	9.93×10^{-4}	56922	Methylcrotonyl-CoA carboxylase subunit 1	Catalyzes the carboxylation of 3-methylcrotonyl-CoA to form 3-methylglutaconyl-CoA
HEXB	0.394	9.95×10^{-4}	3074	Hexosaminidase subunit β	Catalyzes the degradation of ganglioside GM2 and other molecules containing terminal N-acetyl hexosamines
PKM	0.388	0.001	5315	Pyruvate kinase M1/2	Catalyzes the transfer of a phosphoryl group from phosphoenolpyruvate to ADP, generating ATP and pyruvate
RTRAF	0.373	0.002	51637	RNA transcription, translation and transport factor	Involved in negative regulation of protein kinase activity. Regulation of transcription by RNA polymerase II and tRNA splicing
SNX2	0.358	0.003	6643	Sorting nexin 2	Protein sorting in the endocytic pathway
SEC62	0.352	0.003	7095	SEC62 homolog, preprotein translocation factor	Component of the protein translocation apparatus of the endoplasmic reticulum membrane. Post-translational protein translocation into the ER
PIGK	0.350	0.003	10026	Phosphatidylinositol glycan anchor biosynthesis class K	Glycosylphosphatidylinositol (GPI)-anchor biosynthesis
PFKM	0.350	0.003	5213	Phosphofructokinase, muscle	Catalyzes the phosphorylation of fructose-6-phosphate to fructose-1,6-bisphosphate
RAN	0.331	0.005	5901	Ras-related nuclear protein	Small GTP-binding protein belonging to the RAS superfamily. Essential for the translocation of RNA and proteins through the nuclear pore complex. Involved in control of DNA synthesis and cell cycle progression
LGALS3BP	0.331	0.005	3959	Galectin 3-binding protein	β-galactoside-binding protein implicated in modulating cell-cell and cell-matrix interactions
CIP2A	0.330	0.005	57650	Cancerous inhibitor of PP2A	Regulation of neural precursor cell proliferation and spermatogenesis
MFI2	0.330	0.005	4241	Melanotransferrin	Iron-binding properties
CD109	0.327	0.006	135228	150 KDa TGF-β-1-binding protein	Negative regulation of transforming growth factor β signaling
AP2A1	0.327	0.006	160	Adaptor-related protein complex 2 subunit α 1	Subunit of the adaptor protein 2 (AP-2) complex found in clathrin coated vesicles
IGFBP2	0.326	0.005	3485	Insulin-like growth factor binding protein 2	Binds insulin-like growth factors I and II
SYNM	0.323	0.006	23336	Synemin	Cytoskeletal protein that confers resistance to mechanical stress
UQCRB	0.320	0.007	7381	Ubiquinol cytochrome C reductase-binding protein	Electron transfer upon ubiquinone binding. Role in hypoxia-induced angiogenesis through mitochondrial reactive oxygen species-mediated signaling
OPA1	0.317	0.007	4976	OPA1 mitochondrial dynamin-like GTPase	Regulation of mitochondrial stability and energy output
ATP11A	-0.463	1.12×10^{-4}	23250	ATPase phospholipid transporting 11A	Tansport of calcium ions across membranes
DFFA	-0.461	1.21×10^{-4}	1676	DNA fragmentation factor, 45kDa, α polypeptide	DFFA is a substrate for caspase-3 and triggers DNA fragmentation during apoptosis
PSMA6	-0.436	2.82×10^{-4}	5687	Proteasome 20S subunit α 6	Degradation of peptides and proteins
LDHB	-0.431	3.25×10^{-4}	3945	Lactate dehydrogenase B	Catalyzes the interconversion of pyruvate and lactate with concomitant interconversion of NADH and NAD+ in a post-glycolysis process
FAM213A	-0.430	3.38×10^{-4}	84293	Peroxiredoxin-like 2A	Antioxidant activity. Regulation of osteoclast differentiation
RPLP0	-0.429	3.55×10^{-4}	6175	Ribosomal protein lateral stalk subunit P0	Component of the 60S subunit
RBM26	-0.427	3.72×10^{-4}	64062	RNA-binding motif protein 26	involved in mRNA processing

PSMA4	-0.420	4.71×10^{-4}	5685	Proteasome 20S subunit α 4	Degradation of peptides and proteins. Involved in mitotic G1 and G1/S phase transition
TIA1	-0.419	4.74×10^{-4}	7072	Subunit of mitochondrial ATP synthase	
APEX1	-0.415	5.40×10^{-4}	328	Apurinic/apyrimidinic endodeoxyribonuclease 1	DNA repair enzyme with apurinic/apyrimidinic (AP) activity
ATP5F1	-0.410	6.34×10^{-4}	515	ATP synthase peripheral stalk-membrane subunit B	
HNRNPC	-0.409	6.48×10^{-4}	3183	Heterogeneous nuclear ribonucleoprotein C	Involved in pre-mRNA processing
CALM1	-0.406	7.01×10^{-4}	801	Calmodulin 1	Calcium-binding protein. Calcium-induced activation of calmodulin regulates and modulates the function of cardiac ion channels
AHSA1	-0.405	7.26×10^{-4}	10598	Activator of HSP90 ATPase activity 1	Hsp90 protein binding activity. Involved in regulation of ATPase activity
IPO5	-0.402	8.09×10^{-4}	3843	Importin 5	Nucleocytoplasmic transport
CBX5	-0.401	8.21×10^{-4}	23468	Chromobox homolog 5 (HP1 α homolog, *Drosophila*)	Involved in the formation of functional kinetochore through interaction with essential kinetochore proteins
PFAS	-0.399	8.73×10^{-4}	5198	Phosphoribosylformylglycinamidine synthase	*De novo* pathway of purine biosynthesis
ERH	-0.393	0.001	2079	ERH mRNA splicing and mitosis factor	Methyl-CpG binding activity. Involved in pyrimidine nucleoside metabolic processes
RPS13	-0.386	0.001	6207	Ribosomal protein S13	Functions at early steps in ribosome assembly
ZNF428	-0.384	0.001	126299	Zinc finger protein 428	Metal ion-binding activity
